# HacA-Independent Functions of the ER Stress Sensor IreA Synergize with the Canonical UPR to Influence Virulence Traits in *Aspergillus fumigatus*

**DOI:** 10.1371/journal.ppat.1002330

**Published:** 2011-10-20

**Authors:** Xizhi Feng, Karthik Krishnan, Daryl L. Richie, Vishukumar Aimanianda, Lukas Hartl, Nora Grahl, Margaret V. Powers-Fletcher, Minlu Zhang, Kevin K. Fuller, William C. Nierman, Long Jason Lu, Jean-Paul Latgé, Laura Woollett, Simon L. Newman, Robert A. Cramer, Judith C. Rhodes, David S. Askew

**Affiliations:** 1 Department of Pathology & Laboratory Medicine, University of Cincinnati College of Medicine, Cincinnati, Ohio, United States of America; 2 Unité des Aspergillus, Institut Pasteur, Paris, France; 3 Department of Immunology & Infectious Diseases, Montana State University, Bozeman, Montana, United States of America; 4 Division of Biomedical Informatics, Cincinnati Children's Hospital Research Foundation, Cincinnati, Ohio, United States of America; 5 The J. Craig Venter Institute, Rockville, Maryland, United States of America; 6 Department of Medicine, University of Cincinnati College of Medicine, Cincinnati, Ohio, United States of America; Washington University School of Medicine, United States of America

## Abstract

Endoplasmic reticulum (ER) stress is a condition in which the protein folding capacity of the ER becomes overwhelmed by an increased demand for secretion or by exposure to compounds that disrupt ER homeostasis. In yeast and other fungi, the accumulation of unfolded proteins is detected by the ER-transmembrane sensor IreA/Ire1, which responds by cleaving an intron from the downstream cytoplasmic mRNA HacA/Hac1, allowing for the translation of a transcription factor that coordinates a series of adaptive responses that are collectively known as the unfolded protein response (UPR). Here, we examined the contribution of IreA to growth and virulence in the human fungal pathogen *Aspergillus fumigatus*. Gene expression profiling revealed that *A. fumigatus* IreA signals predominantly through the canonical IreA-HacA pathway under conditions of severe ER stress. However, in the absence of ER stress IreA controls dual signaling circuits that are both HacA-dependent and HacA-independent. We found that a Δ*ireA* mutant was avirulent in a mouse model of invasive aspergillosis, which contrasts the partial virulence of a Δ*hacA* mutant, suggesting that IreA contributes to pathogenesis independently of HacA. In support of this conclusion, we found that the Δ*ireA* mutant had more severe defects in the expression of multiple virulence-related traits relative to Δ*hacA*, including reduced thermotolerance, decreased nutritional versatility, impaired growth under hypoxia, altered cell wall and membrane composition, and increased susceptibility to azole antifungals. In addition, full or partial virulence could be restored to the Δ*ireA* mutant by complementation with either the induced form of the *hacA* mRNA, *hacA* ^i^, or an *ireA* deletion mutant that was incapable of processing the *hacA* mRNA, *ireA* ^Δ10^. Together, these findings demonstrate that IreA has both HacA-dependent and HacA-independent functions that contribute to the expression of traits that are essential for virulence in *A. fumigatus*.

## Author Summary

*Aspergillus fumigatus* is the predominant mold pathogen of humans, responsible for life-threatening infections in patients with depressed immunity. The fungus is highly adapted for secretion, a feature that it uses to extract nutrients from the host environment. High rates of protein secretion can overwhelm the protein folding capacity of the endoplasmic reticulum (ER). The resulting ER stress is alleviated by the unfolded protein response (UPR), a signaling pathway that is triggered by the ER-membrane sensor IreA and executed by the downstream transcription factor HacA. This paper uncovers a novel role for IreA in the expression of multiple adaptive traits that allow the fungus to cope with stress conditions that are encountered during infection. Gene expression profiling of Δ*ireA* and Δ*hacA* mutants revealed that IreA signals predominantly through the canonical IreA-HacA UPR pathway under extreme conditions of ER stress, but has unexpected HacA-dependent and HacA-independent functions even in the absence of ER stress. These findings establish IreA as an important regulator of *A. fumigatus* pathogenicity and suggest that therapeutic targeting of the dual functions of this protein could be an effective antifungal strategy.

## Introduction

Approximately one third of the eukaryotic proteome is dedicated to secreted and membrane proteins, making the secretory pathway one of the most active biosynthetic processes in the cell. Many intracellular eukaryotic pathogens use the secretory system for the expression of virulence factors that are crucial for pathogenesis, including adhesion, motility, host cell invasion or the co-opting of host cellular processes [1], [2]. By contrast, the filamentous fungal pathogen *Aspergillus fumigatus* is predominantly extracellular, with no known virulence factors that are specialized derivatives of the secretory pathway. Nevertheless, a highly developed secretory system is an important virulence attribute of this organism because it provides a mechanism for the delivery of hydrolytic enzymes and membrane transporters into, and across, the membrane, which is essential for nutrient acquisition from the host [3]. Many of these enzymes are responsible for damaging host tissues, which contributes to the high mortality rates associated with *A. fumigatus* infections [4].

Protein secretion begins in the endoplasmic reticulum (ER), an extensive membrane network that provides a segregated compartment for the precise folding, modification and export of extracellular and membrane proteins. The ability of this organelle to meet the demand for secretion is limited by the level of ER-resident chaperones, foldases and other modifying enzymes that assist in protein folding [5]. Thus, misfolded proteins can accumulate when the demand for secretion exceeds the protein folding capacity of the ER. Misfolded proteins are prone to non-specific interactions with other molecules in the crowded intracellular environment, resulting in nonfunctional protein aggregates that disrupt ER homeostasis [6].

The unfolded protein response (UPR) is an intracellular signaling pathway that buffers fluctuations in ER homeostasis by increasing ER folding capacity whenever abnormal proteins accumulate in the ER [7]. In fungi, most of what is known about UPR signaling comes from studies in *Saccharomyces cerevisiae* [8]. Upstream control of the yeast UPR is mediated by Ire1p, a type I membrane protein that has an ER lumenal sensing domain and a bifunctional cytosolic tail comprised of a protein kinase domain linked to an endoribonuclease domain [9]. In the absence of ER stress, the lumenal domain is complexed with the ER-resident chaperone BiP, which helps to maintain Ire1 in an inactive state [10]. As unfolded proteins accumulate, due to either adverse environmental conditions or periods of intense protein secretion, BiP dissociates from Ire1 to assist with protein folding. This is followed by high-order oligomerization of Ire1 in the ER membrane, which allows for trans-autophosphorylation and activation of the C-terminal endoribonuclease domain [11], [12], [13]. The only known target of Ire1 endoribonuclease activity is a cytoplasmic mRNA known as *HAC1* in *S. cerevisiae, hacA* in filamentous fungi and *XBP1* in humans [14], [15]. Once activated, the endoribonuclease domain of Ire1 cleaves an unconventional intron from the *HAC1* mRNA, producing a frame-shift that directs the translation of the bZIP transcription factor Hac1p that serves as the master transcriptional regulator of the UPR. After translocating to the nucleus, Hac1p restores the biosynthetic capability of the secretory pathway by upregulating the expression of ER chaperones and foldases and enhancing the degradation of proteins that ultimately fail to fold accurately [14].

In contrast to higher eukaryotes, which possess at least three proximal ER stress sensors, the only known sensor in fungi is Ire1/IreA [8]. The current paradigm of UPR signaling in *A. fumigatus* follows the single linear model established in yeast, in which IreA coordinates the splicing of the uninduced *hacA*u mRNA into its induced form, *hacA*i. Here, we demonstrate an expanded functional scope for IreA in *A. fumigatus*, involving both HacA-dependent and HacA-independent pathways. In addition, we establish a novel role for IreA as a central regulator of virulence, coordinating the expression of multiple virulence-related attributes that collectively support the fitness of *A. fumigatus* in the host environment.

## Results

### Deletion of *A. fumigatus ireA*

The *ireA* cDNA was cloned in two overlapping fragments from reverse-transcribed RNA. A comparison of the genomic and cDNA sequences revealed a 3.4 kb gene with a single intron that would encode a protein of 1,144 amino acids. Structural predictions for the IreA protein revealed a similar domain organization to that of *S. cerevisiae*, Ire1p, including a signal sequence of 27 amino acids, a 477 amino acid ER lumenal domain, a 19 amino acid transmembrane domain and a 621 amino acid cytoplasmic C-terminal region (Figure S1). The cytoplasmic region, which is the most conserved segment of the protein between genera, contains a predicted serine-threonine protein kinase linked to a kinase extension nuclease (KEN) domain [16], the latter of which provides the endoribonuclease activity that is required for regulated splicing of the *HAC1* mRNA in *S. cerevisiae* [17].

In contrast to *Aspergillus niger*, in which the *ireA* gene appears to be essential [18], we found that a Δ*ireA* mutant of *A. fumigatus* is viable. The *ireA* gene was deleted from *A. fumigatus* by replacing the entire coding region with a phleomycin resistance cassette and homologous integrants were identified by genomic Southern blot analysis (Figure S2). Loss of the *ireA* gene had the expected effect of increasing sensitivity to agents that cause acute ER stress by disrupting protein folding, such as tunicamycin (Figure S3).

### Identification of a Core Inducible UPR That Depends on the Canonical IreA-HacA Pathway

**Figure 1:**
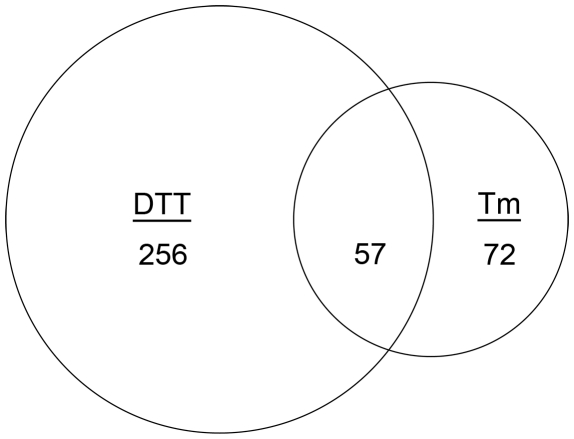
Identification of the core inducible UPR of *A. fumigatus*. Venn diagram demonstrating the overlap between the number of transcripts with increased abundance in the presence of DTT and those that show increased abundance in the presence of Tm. The area of the circles is scaled to the number of transcripts and the values represent distinct counts in each category.

Studies of differentially expressed genes during secretion stress have been previously performed in a number of filamentous fungal species [19], [20], [21]. However, these studies were performed on the wild type (wt) organism, so the specific contributions of IreA or HacA could not be evaluated. To address this, a genome-wide expression profile was generated for wt *A. fumigatus* in the presence of acute ER stress and compared to that of the two mutant strains that are deficient in UPR signaling, Δ*hacA* [15] and Δ*ireA*. Acute ER stress was accomplished by treating the fungus with dithiothreitol (DTT) or tunicamycin (Tm), each of which induces the UPR but through different mechanisms; DTT unfolds proteins by interfering with disulfide bond formation and tunicamycin inhibits the N-linked glycosylation that is necessary for proper protein folding [14]. Because high concentrations of DTT (20 mM for 2 h) have been shown to induce changes in gene expression that are unrelated to the UPR [20], we used a mild DTT treatment (1 mM DTT for 1 h) to minimize these non-UPR effects. Nevertheless, DTT treatment of wt *A. fumigatus* induced changes in mRNA abundance that were higher in both magnitude and scope than treatment with Tm, similar to what has been reported elsewhere (Figure 1) [19], [20].

We employed three criteria to define UPR-regulated genes under conditions of acute ER stress. First, since *hacA* is a known target of the UPR [22], and its mRNA increased in abundance at least 1.5-fold when treated with either DTT or Tm in this study, we set 1.5-fold as the threshold for differential expression (up or down). The resulting data revealed that 256 mRNAs were differentially expressed following treatment with DTT (but not Tm), while 72 mRNAs showed altered abundance following Tm treatment (but not by DTT) (Figure 1). Second, to maximize the detection of UPR-regulated genes, and avoid the identification of genes that are influenced by chemical-specific off-target effects, the dataset was restricted to the 57 mRNAs with altered abundance following both DTT and Tm treatment (Figure 1). Third, mRNAs that failed to change in both the *ΔhacA* and Δ*ireA* mutants in response to DTT and Tm treatment, but did change in the wt strain under these conditions, were considered dependent on both IreA and HacA for differential expression, thus defining them as components of the canonical IreA-HacAi UPR pathway. The data revealed that the vast majority (95%) of the 57 DTT/Tm-responsive mRNAs identified above were unaffected by DTT/Tm treatment in the Δ*hacA* and Δ*ireA* mutants, demonstrating that *A. fumigatus* responds to extreme conditions of ER stress predominantly through the IreA-HacAi pathway. Of these 57 mRNAs, 45 increased in abundance under acute ER stress (Table 1), suggesting that they represent the core components of an inducible UPR (i-UPR) in *A. fumigatus*.

**Table 1:** Functional classification of genes with altered mRNA abundance under conditions of acute ER stress (treatment with DTT or Tm).

Locus	Common Name	HM	NN	DTT	Tm
Increased in response to acute ER stress					
**Protein folding**					
Afu2g04620	ER Hsp70 chaperone bip, putative	*	*	3.4674	2.0488
Afu4g12850	Calnexin	*	*	2.8761	1.7910
Afu1g15050	Hsp70 chaperone (Orp150), putative	*	*	2.8347	1.6695
Afu2g06150	Disulfide isomerase, putative (PdiI)	*	*	2.6996	1.9674
Afu1g05320	Disulfide isomerase, putative	*	*	2.5326	1.8063
Afu3g05400	Dnaj and TPR domain protein	*	*	2.3187	1.4339
Afu2g08300	Dnaj domain protein, putative	*	*	2.0197	1.2698
Afu8g05140	Similar to *A. niger* endoplasmic oxidoreductin-1			1.6820	0.8498
Afu4g07650	Peptidyl-prolyl cis-trans isomerase (CypB), putative	*	*	1.3775	1.2929
Afu6g06610	Dnaj domain protein	*	*	1.2363	0.7329
**ER glycosylation**					
Afu5g08970	Oligosaccharyl transferase subunit (beta), putative	*	*	1.9009	1.1636
Afu6g04210	Mannosyl-oligosaccharide glucosidase, putative	*	*	1.6985	1.4292
Afu7g04110	Glucosidase II β subunit, putative			1.3932	0.6498
Afu2g06280	Oligosaccharyl transferase subunit (gamma), putative	*	*	1.2305	1.0611
Afu3g03020	Phosphoglucomutase, putative			1.1070	0.8079
Afu8g04430	Oligosaccharyl transferase subunit (Stt3), putative		*	1.1014	0.9783
Afu8g04500	Mannosyltransferase PMTI			0.8951	0.6043
Afu7g02180	UDP-N-acetylglucosamine pyrophosphorylase			0.7524	0.7004
**ER-associated degradation**					
Afu8g04840	RING finger protein (Hrd1)	*	*	1.3837	0.5951
**ER translocation/signal peptidase complex**					
Afu5g08130	Protein transport protein SEC61 alpha subunit, putative		*	1.4822	0.6233
Afu5g03220	Microsomal signal peptidase subunit (gp23), putative	*	*	1.4452	0.9169
Afu3g12840	Signal peptidase I	*	*	1.2851	0.9221
Afu8g04260	Translocation protein (Sec66), putative	*	*	1.1657	0.7410
Afu3g08350	Sec20 family		*	0.7147	0.6255
**Cellular transport/vesicle trafficking**					
Afu1g11770	COPII-coated vesicle protein surf4/Erv29			2.1008	0.9775
Afu2g01530	COPII-coated vesicle protein (Erv41)	*	*	2.0185	1.1279
Afu1g05120	COPII-coated vesicle membrane protein Erv46		*	1.5217	0.6556
Afu6g12830	Protein transport protein Sec24, putative			0.8300	0.6094
Afu1g15860	Coatomer subunit delta, putative		*	0.8213	0.6767
**Membrane-associated**					
Afu3g07290	SD08430p			1.7175	0.9538
Afu6g06740	Endoplasmic reticulum calcium ATPase, putative		*	1.6655	1.0729
Afu5g01960	Inorganic phosphate transporter (Pho88)		*	1.3476	0.6568
Afu1g05440	UDP-Glc/Gal ER nucleotide sugar transporter			1.1112	1.0565
Afu2g17930	Bifunctional sterol desaturase/short chain dehydrogenase	*	*	1.0877	0.5944
Afu7g06570	Vacuolar membrane zinc transporter (Zrc1)		*	0.7270	−0.6140
**Transcriptional regulation**					
Afu5g00720	Acetyltransferase, GNAT family family			2.8678	1.6073
Afu3g04070	bZIP transcription factor (HacA)		*	1.4814	0.6420
Afu4g01470	C6 domain protein/fungal-specific transcription factor			0.7889	0.5947
**Amino acid metabolism**					
Afu1g06150	L-serine dehydratase, putative		*	1.0856	0.9025
Afu7g06540	Threonine aldolase, putative			0.8491	0.6323
**Lipid metabolism**					
Afu4g13070	Alpha/beta hydrolase, putative			1.7477	0.7507
**Unclassified**					
Afu6g00690	Conserved hypothetical protein	*	*	1.7940	1.8562
Afu6g00680	Hypothetical protein			1.1532	1.1835
Afu6g04410	DUF1183 domain protein	*	*	0.7494	0.7362
Afu7g00370	Hypothetical protein	*	*	0.7158	0.6925
**Decreased in response to acute ER stress**					
Afu5g02330	Major allergen Asp F1 (Ribotoxin)	*	*	−2.1698	−1.7575
Afu1g13550	Hypothetical protein			−1.2010	−1.0487
Afu2g02310	Sur7 protein, putative	*	*	−0.9534	−0.6468
Afu2g03510	Pheromone processing carboxypeptidase (Sxa2), putative	*	*	−0.8865	−0.9372
Afu5g13100	Hypothetical protein			−0.8757	−0.9398
Afu7g05730	Dihydrolipoamide acetyltransferase component of pyruvate dehydrogenase, putative			−0.8134	−0.7973
Afu4g01290	Endo-chitosanase, pseudogene	*	*	−0.7462	−1.5285
Afu7g05580	Hypothetical protein			−0.6937	−0.5871
Afu4g14000	Tripeptidyl peptidase A	*	*	−0.6800	−0.7639
Afu5g09860	Esterase, putative			−0.6456	−0.7026
Afu7g03970	Hypothetical protein	*	*	−0.6233	−0.6071
Afu2g09290	Antigenic mitochondrial protein HSP60, putative			−0.6045	−0.7077

Differentially regulated transcripts were defined as having a fold-change greater than the arbitrary thresholds of plus and minus 1.5. Values represent log_2_[wt+DTT/wt-DTT] or log_2_[wt+Tm/wt+DMSO vehicle].

*Signal peptide predicted by the hidden markov (HM) or neural network models (NN).

Most of the i-UPR proteins contain predicted signal peptide sequences, consistent with membrane association or secretion functions (Table 1). The largest group contains proteins with functions that are known to facilitate protein folding in the ER, such as chaperones, isomerases and carbohydrate modifying enzymes. As expected from previous studies in other species, most of the genes in the i-UPR dataset affect the secretory pathway at multiple levels and are already known to be downstream of the UPR, such as BiP, Hsp70, protein disulfide isomerase, calnexin and mannosyl-oligosaccharide glucosidase [20], [22], [23]. The presence of these established UPR genes in the i-UPR dataset provides confidence that the microarray hybridization conditions and analysis criteria were appropriate for the identification of UPR genes in *A. fumigatus*.

A small subset of mRNAs decreased in abundance during acute ER stress (Table 1). One of these genes encodes Sur7, a multifunctional transmembrane protein implicated in plasma membrane organization, endocytosis and cell wall biogenesis [24], [25]. A reduction in mRNA encoding Sur7 has also been reported to occur during ER stress in *A. niger* [20]. ER stress in fungi has also been associated with reduced levels of a number of mRNAs encoding secreted proteins, mediated by a pathway known as repression under ER stress (RESS) [26]. The observed decrease in mRNAs encoding tripeptidyl peptidase A, carboxypeptidase and the secreted ribotoxin AspF1 in this study is consistent with the existence of such a mechanism in *A. fumigatus*.

### Identification of a HacA^i^-Independent Gene Regulatory Network Mediated by IreA in the Absence of Acute ER Stress

**Figure 2:**
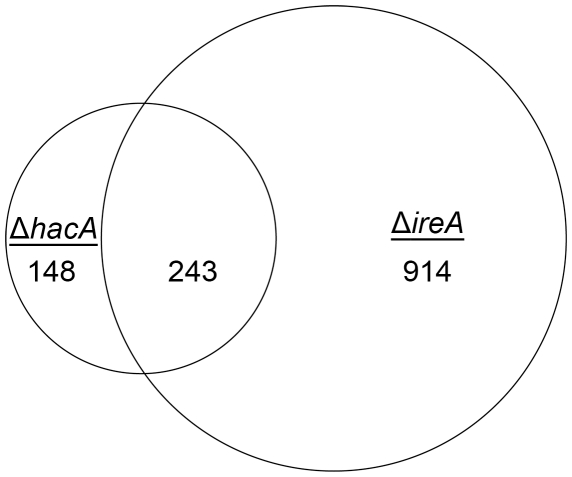
Identification of a HacA-independent gene regulatory network mediated by IreA. Venn diagram demonstrating the overlap between the number of transcripts with decreased abundance in the Δ*hacA* mutant and those that show decreased abundance in the Δ*ireA* mutant relative to wt. The area of the circles is scaled to the number of transcripts and the values represent distinct counts for each category. The list of genes corresponding to each pathway is included in Figure S11.

We found that a substantial amount of *hacA* ^u^ mRNA processing into *hacA* ^i^ could always be detected in wt *A. fumigatus* grown under standard laboratory conditions, suggesting that the IreA-HacA^i^ UPR is active during filamentous growth. To test this, we compared patterns of gene expression under normal growth conditions in the absence of any ER stress-inducing agent. Using a 1.5-fold change in expression level as the cut-off, a total of 1305 mRNAs showed altered abundance in the two mutants, demonstrating a much larger contribution of IreA and HacA to the gene expression signature associated with normal growth than to that associated with acute ER stress (Figure 2). Among these differentially expressed mRNAs, 243 were shared between the Δ*hacA* and Δ*ireA* mutants, suggesting that they are under the control of the canonical IreA-HacA^i^ pathway. Interestingly, only 9 of these mRNAs overlapped with the i-UPR dataset. This suggests that the canonical Ire-HacA^i^ UPR directs a pattern of gene expression that can be broadly divided into a ‘basal’ response and an inducible response, with the basal UPR constituting 80% of the 291 genes in the total IreA-HacA^i^ dataset and the i-UPR representing the remaining 20%. These are likely to represent opposite ends of a spectrum of gene expression that varies in proportion to the level of ER stress, with the basal UPR predominating in the absence of ER stress and the i-UPR dominating under acute ER stress. Gene Ontology (GO) mapping of the basal UPR category revealed enrichment of genes related to mitochondrial function, suggesting that the canonical IreA-HacA^i^ UPR is linked to the regulation of metabolic adaptation during normal filamentous growth (Figure S4).

Since the only known function of Ire1 signaling in yeast is *HAC1* mRNA splicing, a surprising finding from this analysis was that 914 genes had decreased abundance uniquely in the Δ*ireA* mutant, supporting the idea that IreA has functions in filamentous growth that are both broader in scope and independent of HacA. In addition, a small subset of mRNAs (148) were reduced in the Δ*hacA* mutant, but not in the Δ*ireA* mutant, raising the possibility that the predicted protein encoded by the uninduced form of the *hacA* mRNA, HacA^u^, also influences gene expression independently of both HacA^i^ and IreA.

**Figure 3:**
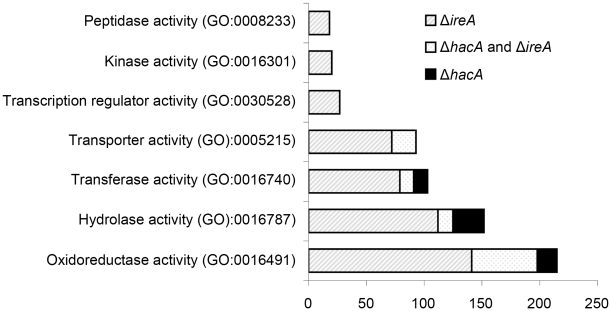
Gene Ontology mapping of differentially expressed genes in Δ*hacA* and Δ*ireA* mutants. Graphical representation of selected multi-level GO categories (from the parent group of Molecular Function) among genes that showed decreased abundance in the Δ*ireA* and Δ*hacA* mutants. The genes were functionally categorized using *A. fumigatus* annotations obtained from BLAST2GO functional annotation repository (taxa ID: 330979). The dataset is limited to genes that were assigned to a GO category at the time of writing and contains 258 genes in the Δ*hacA* dataset and 779 genes belonging to the Δ*ireA* dataset.

GO mapping was performed on the list of genes that showed dependence on HacA and/or IreA for expression (Figure 3). The results demonstrated that the two pathways control a similar proportion of genes in the oxidoreductase category, which is attributed mainly to genes involved in the mitochondrial respiratory chain (Figure S4). Genes with functions in transcriptional regulation and kinase activity were also abundant in the IreA dataset but were conspicuously absent from the HacA^i^ dataset, consistent with the notion that IreA has HacA^i^-independent functions that may connect with other intracellular pathways. Although hydrolases, transferases and transporters were enriched in both groups, their inferred functions were much broader in scope in the IreA group. For example, peptidases, representing a sub-group of all hydrolases, were limited to the IreA subset, further supporting the existence of IreA functions that do not entirely overlap with those of HacA^i^.

A pathway-based enrichment analysis using the Kyoto Encyclopedia of Genes and Genomes (KEGG) database revealed that oxidative phosphorylation was over-represented among mRNAs with decreased abundance in both Δ*hacA* and Δ*ireA* (Table 2). This supports a link between UPR signaling and mitochondrial function in *A. fumigatus*, similar to the recently reported cross-talk between the ER and mitochondrial compartments during ER stress in mammalian cells [27]. N-linked glycosylation showed the greatest enrichment among mRNAs with decreased abundance in Δ*hacA*, consistent with the prominent role of N-linked glycosylation in the maturation of secretory proteins [28]. Steroid biosynthesis and taurine metabolism were also enriched in the dataset of reduced mRNAs in Δ*ireA*, suggesting potential defects in membrane homeostasis and nutritional versatility.

**Table 2:** Over-represented KEGG pathways among genes that show decreased abundance in Δ*hacA* or Δ*ireA* under standard growth conditions.

KEGG pathway		p-value
**Δ** ***hacA***		
Oxidative phosphorylation	afm00190	2.2×10^−03^
N-Glycan biosynthesis	afm00510	3.9×10^−02^
**Δ** ***ireA***		
Oxidative phosphorylation	afm00190	4.4×10^−03^
Steroid biosynthesis	afm00100	1.8×10^−03^
Taurine and hypotaurine metabolism	afm00430	6.7×10^−03^

### HacA^i^-Independent Functions of IreA Synergize with the Canonical UPR to Support Virulence

**Figure 4:**
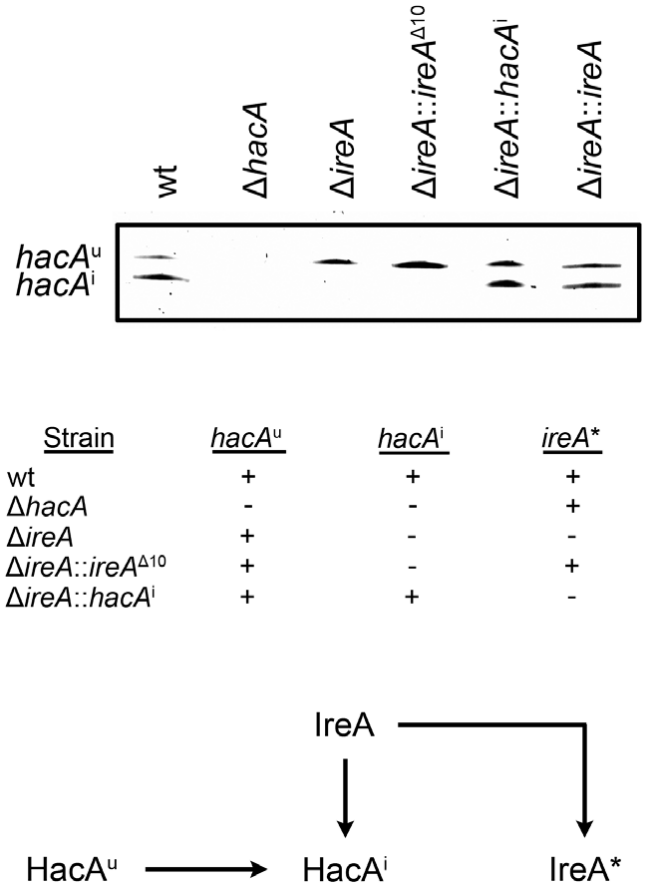
Analysis of *hacA* mRNA splicing in *hacA* and *ireA*-deficient mutants. The *hacA* mRNA was amplified by RT-PCR using primers that span the 20 nucleotide unconventional intron and PCR products were separated on a denaturing acrylamide gel (top figure). The Δ*ireA* and the endoribonuclease-deficient Δ*ireA*::*ireA* ^Δ10^ mutants lack the ability to process the *hacA* ^u^ mRNA into *hacA* ^i^. The inability of Δ*ireA* to process *hacA* ^u^ was rescued by transforming Δ*ireA* with the constitutively spliced *hacA* ^i^ cDNA (Δ*ireA*::*hacA* ^i^) or the wt *ireA* gene (Δ*ireA:*:*ireA*). The presence (+) or absence (−) of *hacA/ireA*-dependent functions in these strains is summarized in the table: *hacA* ^u^: IreA-independent functions mediated by the unspliced form of the *hacA* mRNA, *hacA* ^i^: canonical UPR functions mediated by the induced form of the *hacA* mRNA, ireA*: HacA^i^-independent functions of IreA revealed by the microarray RNA analysis in this study. A schematic illustration of the relationship between the pathways described in this study is shown below.

To determine how changes in IreA- and HacA-dependent gene expression influence virulence, the mutants were compared in a mouse model of invasive aspergillosis. Since the Δ*ireA* mutant is deficient in both HacA^i^-dependent and HacA^i^-independent functions of IreA, two additional *ireA* mutant strains were constructed to separate the two functions (Figure 4). First, an endoribonuclease-deficient IreA strain, Δ*ireA*::*ireA* ^Δ10^, was generated by replacing the *ireA* gene with a deletion mutant that is missing 10 conserved amino acids (1076–1085) from the endoribonuclease domain (Figure S1). This domain contains three amino acids that form the essential catalytic center of the endoribonuclease domain of *S. cerevisiae* Ire1p [16]. The Δ*ireA*::*ireA* ^Δ10^ mutant would be expected to lack *hacA* ^u^ mRNA processing capacity, but retain any HacA^i^-independent functions that are not dependent on endoribonuclease activity (Figure 4). Secondly, a spliced *hacA*i expression cassette was introduced into the Δi*reA* mutant to generate a strain that would possess constitutive HacA^i^ signaling but be deficient in HacA^i^-independent functions of IreA. A summary of these strains is shown in Figure 4. As expected, neither the Δ*ireA* nor Δ*ireA*::*ireA* ^Δ10^ mutants were able to process *hacA* ^u^ mRNA into *hacA* ^i^, thereby establishing the dependence of *hacA* ^u^ mRNA processing on the endoribonuclease activity of IreA in *A. fumigatus*. Reconstitution of the Δ*ireA* mutant with either *hacA* ^i^ or *ireA* restored *hacA* ^i^ expression (Figure 4, top panel).

**Figure 5:**
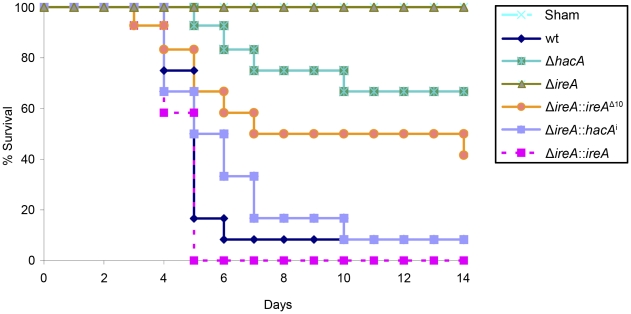
IreA is essential for virulence. Groups of 12 CF-1 outbred mice were immunosuppressed with triamcinolone acetonide and infected intranasally with 2×10^6^ conidia from the indicated strains and mortality was monitored for 14 days. The Δ*ireA* strain was avirulent in this model (p<0.001), which contrasts the partially attenuated virulence of Δ*hacA* (p<0.05) and Δ*ireA*::*ireA* ^Δ10^ (p<0.05). The virulence of the Δ*ireA*::*hacA* ^i^ and Δ*ireA*::*ireA* strains was statistically indistinguishable from wt. The avirulence of Δi*reA* was confirmed in a separate experiment (Figures S5 and S6).

**Figure 6:**
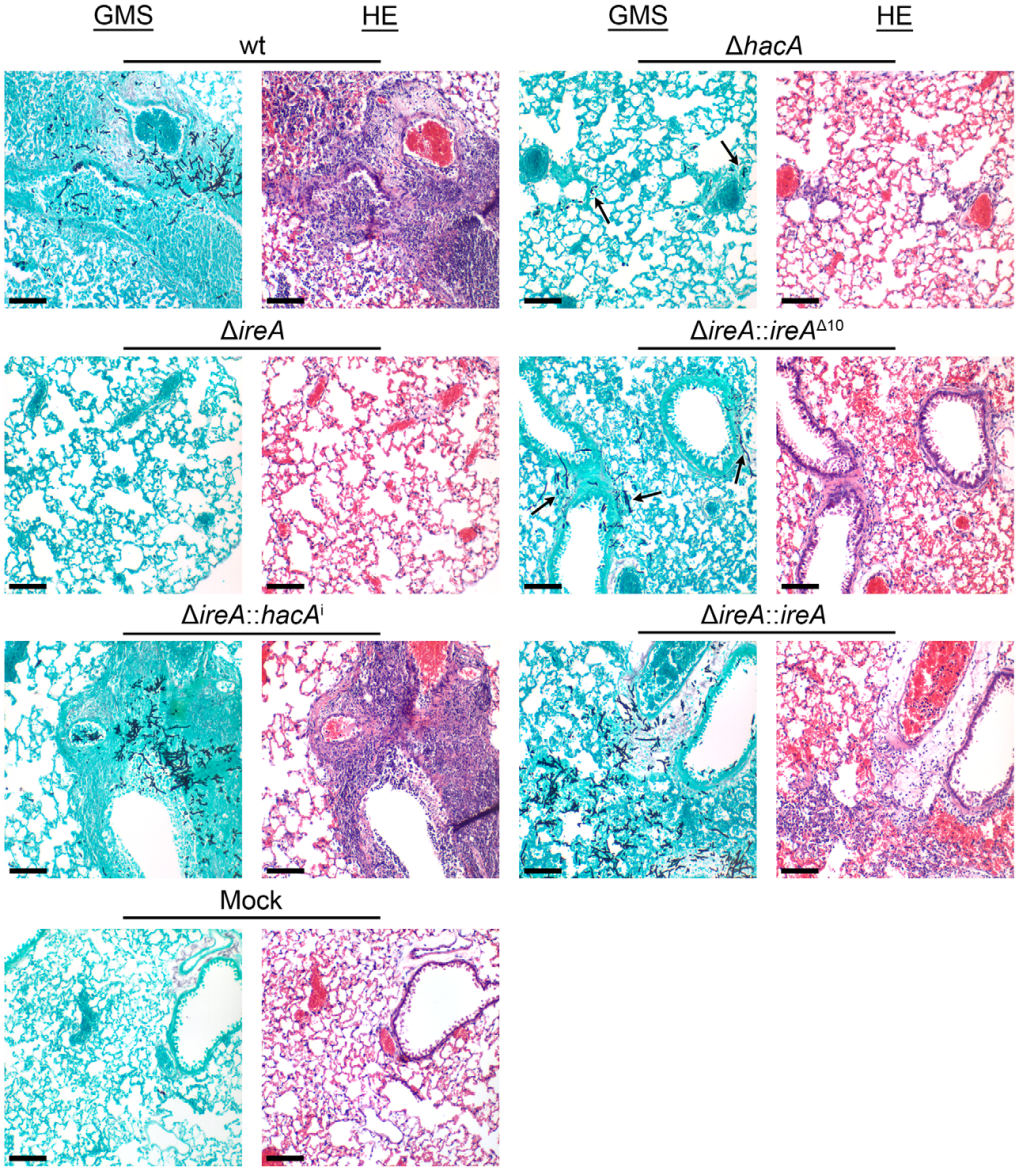
Histopathology of infected lung tissue. Mice were infected as described in Figure 5 and sacrificed on day 3 post-infection. The lungs were sectioned at 5 µm and stained with hematoxylin and eosin (H&E) or Grocott methenamine silver (GMS). Microscopic examinations were performed on an Olympus BH-2 microscope and imaging system using Spot software version 4.6. Scale bar represents 100 µm.

The Δ*ireA* mutant was avirulent, which contrasted the partial virulence of Δ*hacA* (Figure 5), suggesting that IreA contributes to pathogenicity independently of HacA. In support of this conclusion, we found that reconstitution of Δ*ireA* with the *ireA* ^Δ10^ mutant partially restored virulence and reconstitution with a constitutively spliced *hacA* ^i^ gene fully restored virulence. Histopathologic analysis of infected lungs on day 3 post-infection was consistent with these mortality data (Figure 6). Mice infected with the wt, Δ*ireA*::*ireA* and Δ*ireA*::*hacA* ^i^ strains revealed extensive fungal growth surrounded by inflammation and tissue necrosis. However, very little fungal growth or inflammation was observed in the Δ*ireA*-infected mice at the same time point. Although a few swollen conidia could be identified in Δ*ireA*-infected lungs on the day following infection (Figure S6), no viable fungus could be recovered from surviving mice at the end of the experiment (data not shown), indicating that the mice were able to clear the infection. Mice infected with the Δ*ireA*::*ireA* ^Δ10^ and Δ*hacA* strains revealed an intermediate amount of fungal growth (Figure 6, arrows) that was associated with a small amount of inflammation. These data suggest that the canonical IreA-HacA^i^ UPR works together with the HacA^i^-independent functions of IreA to support the virulence of *A. fumigatus*.

### IreA Supports Growth at 37°C

**Figure 7:**
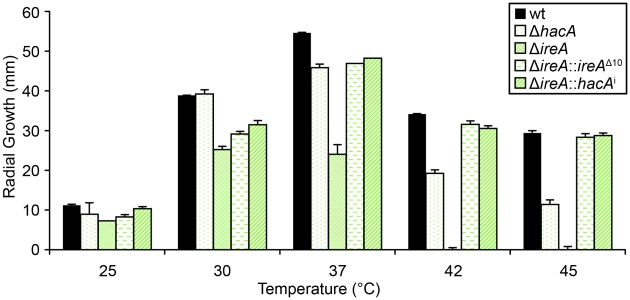
IreA contributes to thermotolerant growth. Equal numbers of conidia from the indicated strains were spotted onto the center of a plate of rich medium (YPD) and radial growth (colony diameter) was measured after 4 days at the indicated temperatures.

The avirulence of the Δ*ireA* mutant suggests that IreA contributes to the expression of adaptive traits that the fungus requires for optimal fitness in the host. We therefore examined the Δ*ireA* mutant for its ability to withstand different types of stress that may be encountered in the host during infection [29]. The ability to grow rapidly at mammalian body temperature is one of the major virulence determinants of *A. fumigatus* [30], [31], [32]. Since higher temperatures induce conformational changes in proteins, and IreA is the major sensor of misfolded proteins in the ER, IreA is uniquely positioned to coordinate adaptive responses to thermal stress. Analysis of growth rates at different temperatures confirmed that IreA promotes growth at 37°C and was essential for growth at 42°C (Figure 7). The Δ*hacA* mutant was also thermosensitive, but to a lesser extent. Interestingly, the expression of *ireA* ^Δ10^ or *hacA* ^i^ in the Δ*ireA* background corrected most, though not all, of the thermosensitivity of Δ*ireA* (Figure 7), indicating that both IreA and HacA contribute to functions that are needed for optimal growth at mammalian body temperature.

### IreA Supports Growth in Hypoxia

**Figure 8:**
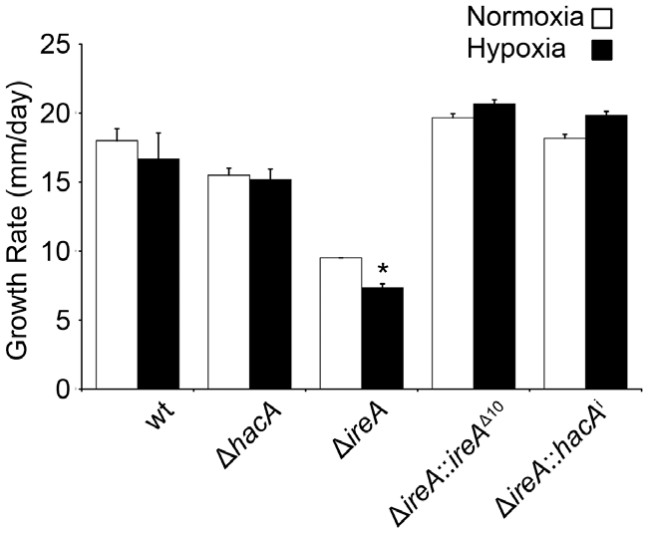
IreA contributes to growth in hypoxia. Equal numbers of conidia were placed in the center of YPD plates and cultured under normoxic (21%) or hypoxic (1% 0_2_) conditions for 4 days at 37°C. The growth rates from triplicate plates were calculated in 24 hour intervals between 48 and 96 hours of incubation and the average growth rate was plotted. The Δ*ireA* mutant was the only strain that showed reduced growth under hypoxia relative to normoxia. *Statistically significant (p<0.001).

*A. fumigatus* encounters areas of limited oxygen availability in the host, and the ability of the fungus to adapt to these hypoxic zones is an established virulence determinant for this organism [33]. Because the UPR has been implicated in hypoxia adaptation in mammalian cells [34], we compared the growth of Δ*hacA* and Δ*ireA* at levels of oxygen that are similar to those encountered in host tissues [33]. As shown in Figure 8, the Δ*ireA* mutant was the only strain that was adversely affected by hypoxia, displaying a 23% reduction in growth rate relative to growth under normoxic conditions. This indicates that IreA supports the fitness of *A. fumigatus* when oxygen tension is low, which may contribute to the observed lack of virulence of Δ*ireA*.

### IreA Supports Cell Wall and Membrane Homeostasis

**Figure 9:**
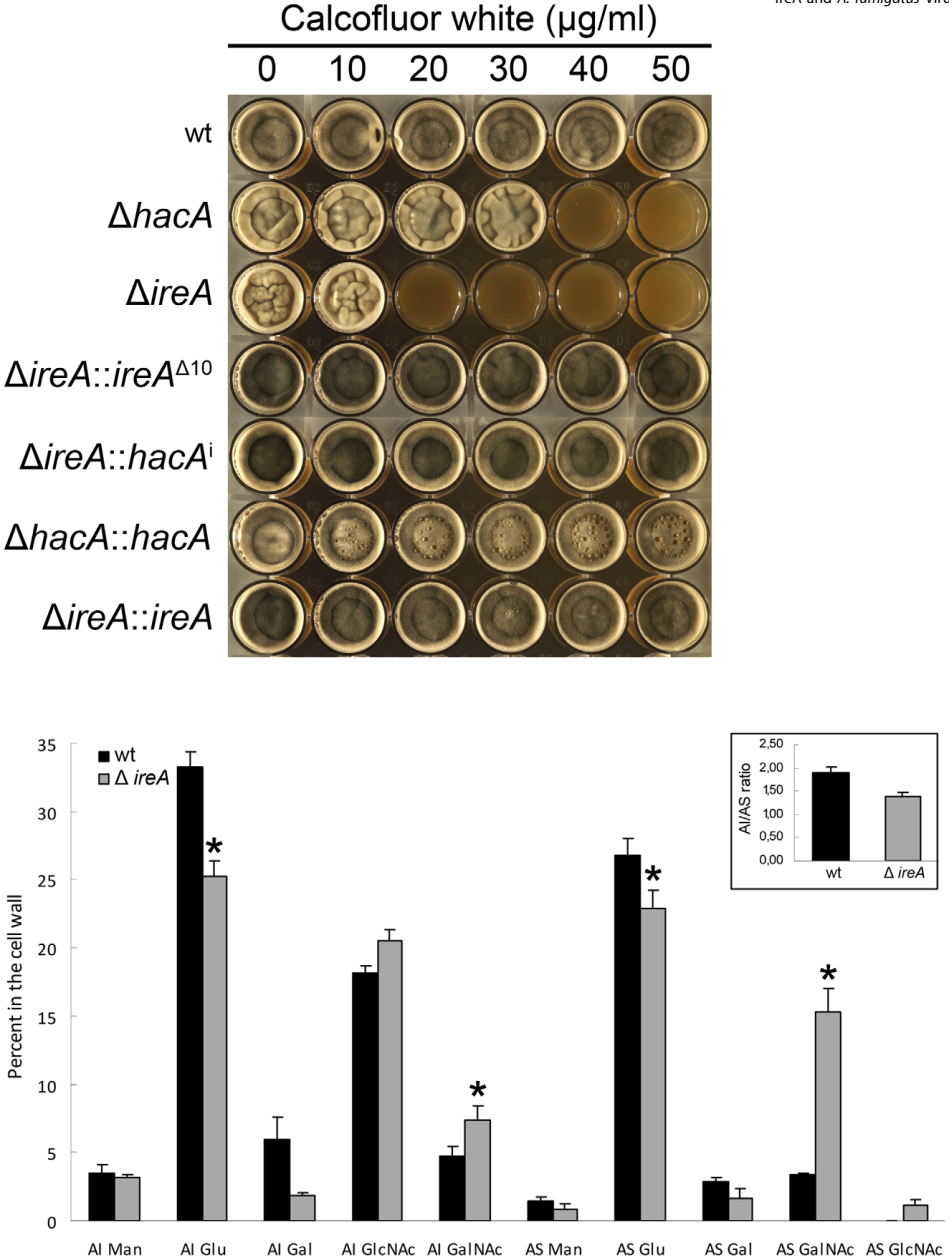
IreA contributes to cell wall homeostasis. Top: Equal numbers of conidia were inoculated into the center of each well of a multi-well plate containing YPD agar supplemented with the indicated concentrations of calcofluor white and incubated for 96 h at 30°C. Below: monosaccharide composition of the alkali-insoluble and alkali-soluble fractions of wt and Δ*ireA* mycelial walls. Results are expressed as the percent of individual monosaccharides in the cell wall. Values represent the average of four replicates ± standard deviation. *Statistically significant (p<0.01). The AI/AS ratio was 1.90±0.1 for wt and 1.39±0.1 for Δ*ireA* (inset).

The cell wall of *A. fumigatus* provides a rigid, yet permeable, barrier that represents the major interface between the fungus and the host environment [35]. We have previously demonstrated that the Δ*hacA* mutant is hypersensitive to cell wall stress, suggesting that the support provided by HacA to the secretory system is important for cell wall homeostasis. Here, we demonstrate that the Δ*ireA* mutant is even more profoundly affected by cell wall stress, showing reduced growth at concentrations of the cell wall damaging agent calcofluor white (CFW) that had minimal effect on the *ΔhacA* mutant (Figure 9, top panel). The expression of *ireA* ^Δ10^ or *hacA* ^i^ in the Δ*ireA* background restored CFW resistance to wt levels.

The *A. fumigatus* cell wall can be divided biochemically into an alkali insoluble fraction comprised of β(1–3)-glucan, chitin and galactomannan, and an alkali soluble fraction containing predominantly α(1–3)-glucan and galactomannan [36]. Analysis of the cell wall monosaccharide composition confirmed that the Δ*ireA* cell wall was abnormal. Decreased glucose was found in the two major cell wall fractions, as well as increased galactose in the alkali insoluble fraction and increased N-acetylgalactosamine in the alkali soluble fraction (Figure 9, bottom panel).

**Figure 10:**
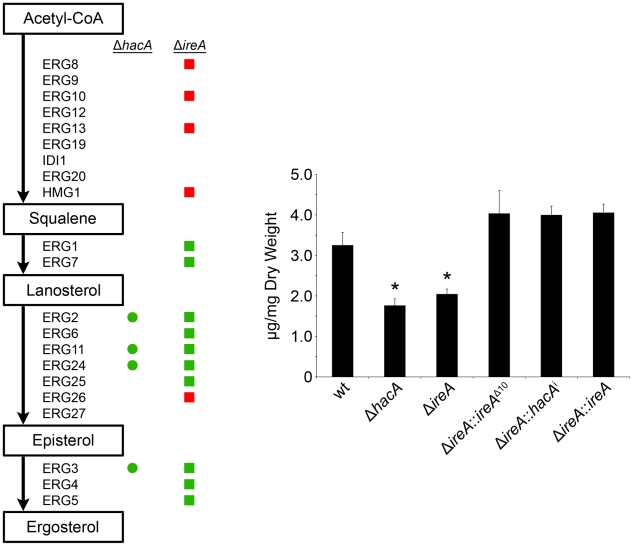
IreA contributes to ergosterol biosynthesis. Left: Schematic representation of genes in the ergosterol biosynthetic pathway that show a ≥1.5-fold decrease (green) or increase (red) in Δ*hacA* (circles) or Δ*ireA* (squares) relative to wt. The ergosterol pathway is derived from *S. cerevisiae*. Right: comparison of ergosterol content. Values represent the average of three replicates, expressed as µg ergosterol per mg dry fungal biomass. *Statistically significant (p<0.05).

Ergosterol is the major sterol in fungal membranes, responsible for controlling membrane fluidity and regulating the distribution of membrane proteins [37]. Our microarray analysis revealed that a number of mRNAs involved in ergosterol biosynthesis [38] had decreased abundance in both Δ*hacA* and Δ*ireA* relative to wt (summarized in Figure 10, full dataset is shown in Figure S11). Analysis of mycelial sterols by gas chromatography revealed decreased ergosterol levels in both strains (Figure 10, right panel), suggesting that the UPR integrates with the ergosterol biosynthetic pathway in *A. fumigatus*. Both strains also had increased sensitivity to azole antifungal drugs, presumably due to further inhibition of ergosterol biosynthesis caused by the inhibitory action of azoles against the ERG11 enzyme (Table 3). A hierarchical clustering of genes in the ergosterol biosynthetic pathway that show differential expression in Δ*hacA* or Δ*ireA* is shown in Figure S7. Interestingly, the Δ*ireA* mutant revealed increased expression of 4 mRNAs that are upstream of squalene in the ergosterol pathway (Figure 10). Although this could be due to compensatory upregulation, the fact that it was not seen in the Δ*hacA* mutant suggests that the loss of IreA has effects on this pathway that are HacA-independent.

**Table 3:** Azole antifungal susceptibility using the Sensititre YeastOne^©^ method.

	MIC (µg/ml)	
	VOR	IZ
	MIC range 0.008 – 4 µg/ml	MIC range: 0.03 – 2 µg/ml
wt	0.5	0.5
Δ*hacA*	0.06	0.12
Δ*ireA*	<0.008	0.06
Δ*ireA*::*ireA^Δ10^*	0.25	0.25
Δ*ireA*::*hacA^i^*	0.25	0.5
Δ*ireA*::*ireA*	0.5	0.5

VOR: voriconazole, IZ: itraconazole (IZ).

### IreA Supports Nutritional Versatility

**Figure 11:**
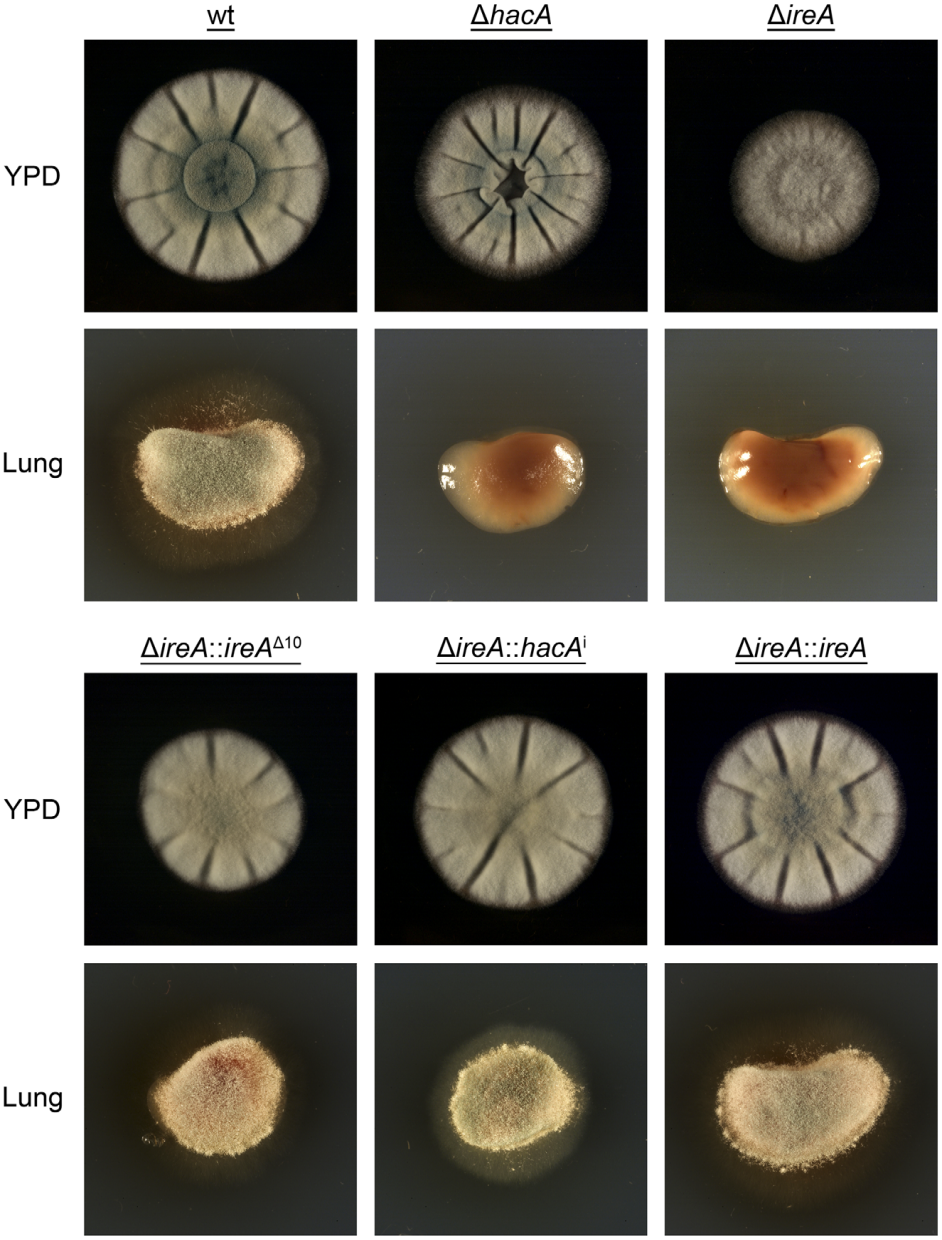
IreA promotes growth on lung tissue. Equal numbers of conidia from the indicated strains were inoculated into the center of a plate of YPD or onto an explant of mouse lung tissue that was placed onto the surface of a plate of 1% agarose in sterile distilled water. The plates were photographed after 2 days of incubation at 37°C.

*A. fumigatus* encounters nutritional stress in the host environment, which requires metabolic reprogramming by the fungus to effectively use the host as a nutrient source [3], [29]. To determine the importance of IreA to nutritional versatility, growth was compared on plates of YPD medium (representing a rich substrate of pre-digested proteins) or on explants of mouse lung tissue (representing an undigested substrate of complex biological material that the fungus encounters during infection). Although the Δ*ireA* mutant was able to grow on YPD medium, it was unable to do so when inoculated onto a lung explant, even after 7 days of incubation (Figure 11). The Δ*hacA* strain was also impaired on lung tissue, as previously reported [15], although some growth could be detected on the surface of the explant when examined microscopically (data not shown).

**Figure 12:**
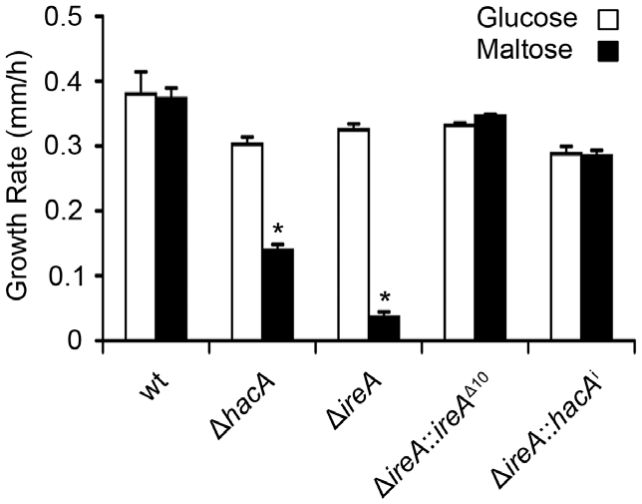
HacA and IreA promote growth on maltose medium. Equal numbers of conidia from the indicated strains were spotted onto plates of *Aspergillus* minimal medium containing either 1% maltose or glucose as the carbon source and growth rate (mm/h) was calculated after 3 days of incubation at 30°C.

All filamentous fungi modulate the activity of the secretory pathway in response to the conditional need for extracellular enzymes. Previous studies have shown that the growth of *A. niger* on the disaccharide maltose elicits a high rate of protein secretion that is accompanied by a transcriptional response resembling the UPR [39]. The Δ*ireA* mutant showed a striking growth defect on maltose relative to growth on a monosaccharide (glucose). This could be rescued by reconstitution with *ireA* ^Δ10^ or *hacA* ^i^, consistent with a role for IreA in the adaptation to maltose-induced secretion stress (Figure 12). The Δ*hacA* mutant was also impaired on maltose, but to a lesser extent than Δ*ireA*.

**Figure 13:**
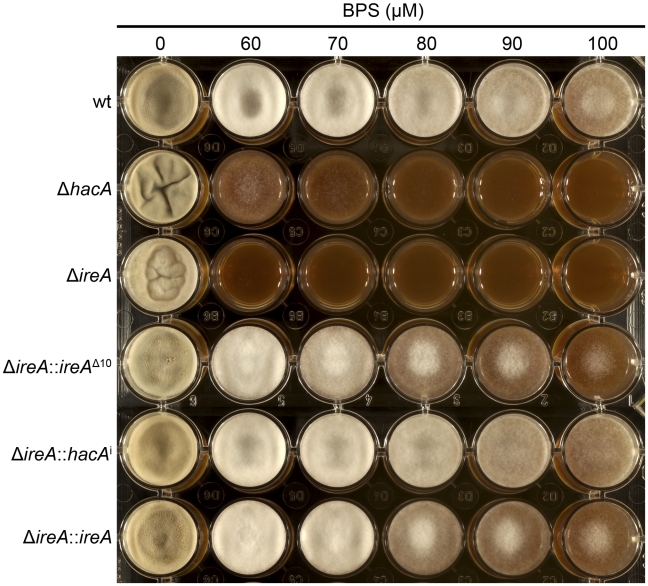
IreA facilitates adaptation to iron starvation. Equal numbers of conidia from the indicated strains were spotted onto YPD medium containing the indicated concentrations of the iron chelator BPS and incubated for 72 h at 30°C. The Δ*hacA* and Δ*ireA* mutants showed increased sensitivity to BPS.

*A. fumigatus* faces iron starvation during infection because the host immune system uses iron-sequestration mechanisms to withhold this essential nutrient from invading microbes [40]. *A. fumigatus* adapts to these conditions by upregulating iron acquisition pathways, which have been shown to be necessary for virulence [41], [42]. Our microarray data showed that at least three mRNAs encoding proteins involved in siderophore-mediated iron acquisition had decreased abundance in Δ*ireA*, as well as components of reductive iron assimilation in both Δ*hacA* and Δ*ireA.* (Figure S11). To determine whether these changes influence iron homeostasis, growth was compared in medium that was rendered iron-deficient by the addition of the iron chelator bathophenanthroline disulfonate (BPS). As shown in Figure 13, the Δ*ireA* mutant was unable to grow in the presence of concentrations of BPS that only partially inhibited Δ*hacA* and had little-to-no effect on wt. The ability to grow in BPS could be rescued by complementation of Δ*ireA* with *ireA* ^Δ10^ or *hacA* ^i^, confirming that IreA has both HacA^i^-dependent and HacA^i^-independent functions that facilitate adaption to iron starvation conditions.

## Discussion

To gain insight into the scope of the UPR in *A. fumigatus* we have compared the genome-wide expression profiles of Δ*hacA* and Δ*ireA* mutants in the presence or absence of ER stress. The data revealed that HacA and IreA collectively influence the expression of over 1300 genes, constituting over 13% of all defined open reading frames in this organism. We found that *A. fumigatus* responds to extreme conditions of ER stress by signaling through the canonical IreA-HacA^i^ pathway, resulting in the activation of a program of gene expression (the i-UPR) that is qualitatively similar to what has been described in yeast [23]. However, we also discovered that the IreA-HacA^i^ UPR was active in the absence of exogenous ER stress, signifying a requirement for basal UPR activity during normal filamentous growth. Since the UPR has been shown to facilitate budding yeast cytokinesis [43], we speculate that *A. fumigatus* requires subtle changes in UPR signaling to buffer dynamic fluctuations in ER stress caused by the constant cell wall remodeling that occurs during hyphal growth [44]. Interestingly, the gene expression profile of the i-UPR during acute ER stress was strikingly different from that of the basal UPR in the absence of ER stress. This suggests that *A. fumigatus* can qualitatively modify the output of the response in proportion to the level of stress, possibly by integrating with other pathways that influence ER homeostasis.

Surprisingly, we found that 70% of the differentially expressed genes in the absence of ER stress were HacA^i^-independent, providing evidence for novel IreA functions during normal filamentous growth. These expanded functions for IreA differ from what has been described in yeast, where the only known function of Ire1 is the activation of the downstream UPR. A possible explanation for this difference is the spatial segregation of function in the interconnected hyphal compartments of a filamentous fungus [45]. This represents an increased level of complexity relative to yeast that may have driven the need for greater flexibility in IreA function. Unique functions for Ire1 have been previously suggested in higher eukaryotes [46], [47]. However, to our knowledge, this is the first report in fungi to demonstrate major regulatory functions for the IreA sensor that go beyond the canonical IreA-HacA^i^ UPR. Interestingly, a small subset of mRNAs showed decreased abundance in the Δ*hacA* mutant, but not in Δ*ireA,* suggesting that the uninduced form of the *hacA* mRNA, *hacA* ^u^, can influence gene expression independently of both HacA^i^ and IreA. It is not yet clear whether this is due to the *hacA* ^u^ mRNA or its encoded product. However, the association of *hacA* ^u^ mRNA with polysomes, together with the high degree of conservation of the predicted HacA^u^ protein among filamentous fungi (data not shown), argues in favor of the translation of *hacA* ^u^ mRNA in *A. fumigatus*.

The changes in gene expression caused by loss of UPR function correlated with a reduction in virulence for the Δ*hacA* strain and a complete loss of virulence for the Δ*ireA* mutant, suggesting that the HacA^i^-independent gene regulatory networks controlled by IreA are functionally entwined with the canonical IreA-HacA^i^ pathway to influence the expression of key virulence attributes. One of these traits is likely to involve thermotolerance. The Δ*ireA* mutant was much more growth impaired than the Δ*hacA* mutant at 37°C, revealing a key function of IreA in the regulation of growth at mammalian body temperature. The reduced ergosterol content of both of these strains may contribute to their inability to tolerate high temperatures. Ergosterol is the major sterol in fungal membranes and, like its mammalian counterpart cholesterol, is responsible for decreasing membrane fluidity by restricting the flexibility of phospholipid acyl chains and limiting permeability to small molecules [37], [48]. Since high temperatures also increase membrane fluidity and permeability [49], the combined effects of reduced ergosterol and thermal stress is likely to disrupt membrane stability and interfere with rapid growth.

Gene expression profiling of *A. fumigatus* during the early stages of infection has suggested that *A. fumigatus* is under nutrient stress in the host environment, requiring upregulation of pathways involved in iron transport and hydrolase secretion to maximize nutrient acquisition from the host [50]. The Δ*ireA* and Δ*hacA* mutants had reduced expression of a number of iron acquisition genes (summarized in the full dataset, Figure S11), which correlated with the reduced growth of both strains under iron limited conditions (Figure 11). Since the ability of *A. fumigatus* to adapt to iron starvation is crucial for pathogenicity [41], the more severe iron starvation defect of Δ*ireA* relative to Δ*hacA* correlates well with the avirulence of Δ*ireA* and the partial virulence of Δ*hacA*. We also found that the Δ*ireA* mutant was more growth impaired than Δ*hacA* when challenged to grow on complex nutrient sources that require secreted hydrolases for nutrient acquisition, providing further support for a role for IreA in the nutritional versatility of this fungus.

A reduction in glucose content was observed in both fractions of the Δ*ireA* mutant cell wall, indicating decreased levels of β(1–3)-glucan and α(1–3)-glucan. This is similar to what was reported in the Δ*hacA* mutant [15], suggesting that it is caused by the loss of IreA-HacA^i^ signaling. A substantial increase in the proportion of N-acetylgalactosamine was found in the Δ*ireA* cell wall, which was not previously seen in the Δ*hacA* mutant [15]. However, the significance of this change is not yet known due the limited understanding of the role of galactose polymers in cell wall homeostasis. It is conceivable that some of these cell wall changes could influence virulence by unmasking carbohydrate epitopes that promote phagocytic clearance by the immune system. However, we found only a slight increase (10–15%) in neutrophil-mediated killing of the gΔ*ireA* mutant relative to wt (data not shown), suggesting that the major virulence defect in this mutant is more likely to be a consequence of poor fitness in the host environment than altered susceptibility to phagocytic killing, particularly in the context of an immunocompromised host.

The UPR has been implicated in hypoxia adaptation in mammalian cells, a function that is attributed to *XBP1* [34]. This contrasts our findings in *A. fumigatus*, where IreA, but not HacA, was required for optimal growth in hypoxia. The growth of Δ*ireA* in limited oxygen was 23% lower than what was observed under normoxic conditions. Although this is a relatively modest reduction when compared to the effects of deleting SrbA, the major regulator of hypoxia adaptation in *A. fumigatus* [33], it is one of multiple defects in the Δ*ireA* mutant that are likely to act synergistically to impair the pathogenic potential of the fungus in the host environment. Since optimum growth under hypoxia requires the mitochondrial respiratory chain [51], the decreased abundance of oxidative phosphorylation mRNAs in the Δ*ireA* mutant may contribute, at least in part, to the observed hypoxia growth defect. SrbA is the ortholog of fission yeast Sre1, an ER membrane-bound protein that monitors sterol synthesis as an indirect measure of oxygen supply [52]. Since IreA and SreA are both ER-membrane proteins that are linked to both ergosterol synthesis and hypoxia adaptation, it is intriguing to speculate that there is cross-talk between the two pathways and experiments to test this possibility are underway.

The findings from this study demonstrate that HacA^u^, HacA^i^ and IreA have independent functions that influence the biology of *A. fumigatus*. The Δ*ireA* mutant lacks two of these functions, mediated by HacA^i^ or the HacA^i^-independent activities of IreA. Reconstitution of Δ*ireA* with either *hacA* ^i^ or *ireA* ^Δ10^ genes restored one of the two pathways, which largely rescued the in vitro phenotypes, suggesting that *A. fumigatus* requires at least two of these three functions to support optimal growth under stress conditions. In addition, we found that partial or full virulence could be restored to the Δ*ireA* mutant by complementation with either *hacA* ^i^ or *ireA* ^Δ10^ genes, demonstrating that virulence is regulated by the HacA^i^-dependent and HacAi-independent functions of IreA. Although the ability of the *hacA* ^i^ gene to fully restore virulence suggests that HacA^i^ signaling was sufficient to restore pathogenicity to Δ*ireA*, an important caveat to this interpretation is that the reconstituted *hacA* ^i^ gene is not under the control of regulated *hacA* mRNA processing, which could increase protein expression and influence virulence. Nevertheless, the fact that reconstitution with *hacA* ^i^ or *ireA* ^Δ10^ was able to restore some virulence potential to the avirulent Δ*ireA* mutant provides strong support for overlapping functions of IreA and HacA in the pathogenicity of *A. fumigatus*.

Overall, the data in this study are consistent with the following model for IreA function in *A. fumigatus*. In the absence of ER stress, IreA coordinates basal HacA^i^ activity to buffer dynamic fluctuations in ER stress that are likely to occur in response to the normal demands of filamentous fungal physiology. Under conditions of severe ER stress, such as a sudden increase in the demand for secretion or exposure to adverse environmental conditions that cause widespread protein folding, IreA increases *hacA* ^u^ mRNA splicing, resulting in the activation of the i-UPR. The pattern of gene expression that characterizes the i-UPR benefits the fungus under extreme conditions because it is more narrowly focused on the secretory pathway than is the basal UPR, allowing for a speedy recovery of ER homeostasis. Although the canonical IreA-HacA^i^ pathway controls both the basal UPR and the i-UPR, it is assisted by complementary signaling networks driven by the HacA^i^-independent functions of IreA, most notably for the expression of traits that are essential for virulence. The precise mechanism by which IreA controls gene expression independently of HacA is not yet known, but an intriguing possibility is that the kinase domain, and/or putative ligand-binding pockets recently identified at the dimer interface of the KEN domain [53], can functionally integrate IreA with other signaling pathways. Regardless of how this is accomplished, the reliance of *A. fumigatus* on IreA for virulence underscores the future potential for targeting the functions of this protein with novel antifungal therapy. Moreover, the recent discovery that HacA is required for virulence of the plant fungal pathogen *Alternaria brassicicola* [54] suggests that targeting the UPR could have broad implications for the control of both human and plant fungal pathogens.

## Materials and Methods

### Strains and Culture Conditions

The *A. fumigatus* strains used in the study are listed in Figure S8. Conidia were harvested from colonies grown on OSM plates (*Aspergillus* minimal medium [55] containing 10 mM ammonium tartrate and osmotically stabilized with 1.2 M sorbitol). Radial growth rates were measured by spotting 5,000 conidia onto the center of a 100 mm plate containing 40 mL of YPD medium (1% yeast extract, 2% peptone, 2% glucose) and monitoring colony diameter daily. YPD was selected because it best supports the growth of the Δ*ireA* mutant. For analysis of cell wall stress response, 2,000 conidia were spotted in a 5 µl volume in each well of a 24-well plate containing YPD supplemented with various concentrations CFW. The plates were incubated at 30°C for 6 days before being photographed. An incubation temperature of 30°C was used wherever possible because it minimized the difference in growth rate between wt and the Δ*ireA* mutant. For analysis of growth in iron-depleted medium, 2,000 conidia were inoculated into YPD medium containing the iron chelator BPS (Sigma #11890) and incubated for 72 h at 30°C. For analysis of growth on lung tissue, explants of mouse lung were placed onto the surface of a plate of 1% agarose in sterile distilled water. The lung tissue was inoculated with 2,000 conidia in a 5 µl volume of sterile water and fungal growth was monitored daily for 7 days at 37°C.

### Hypoxic Cultivation

Normoxic conditions were considered general atmospheric levels within the lab (∼21%). For hypoxia conditions, an INVIVO_2_ 400 Hypoxia Workstation (Ruskinn Technology Limited, Bridgend, UK) was used to maintain an atmosphere of 1% O_2_, 5% CO_2_ and 94% N_2_. Colony growth was quantified as described [33]. Briefly, 5 µl aliquots containing 1×10^6^ conidia from freshly harvested OSM plates were placed onto the center of a plate of YPD and incubated for 4 days under normoxic or hypoxic conditions at 37°C. The experiment was performed in three biological replicates.

### Disruption and Reconstitution of the *A. fumigatus ireA* Gene

All PCR primers used in this study are shown in Figure S9. A complete deletion of the *A. fumigatus ireA* gene (Genbank accession XP_749922) was accomplished using the split-marker approach. The 5′ flank of the *ireA* gene was PCR-amplified from genomic DNA (primers 529 and 530) to create PCR product #1, and the 3′ flank was PCR-amplified with primers 531 and 532 to generate PCR product #2. The phleomycin resistance cassette was PCR amplified into two partially overlapping fragments using primers 398 and 399 to generate PCR product #3 and primers 409 and 410 to generate product #4. Overlap PCR was then used to combine PCR products #1 and #3 into PCR product #5 (primers 529 and 408), and PCR products #2 and #4 into PCR product #6 (primers 410 and 532). PCR products #5 and #6 were then cloned into pCR-Blunt II-TOPO (Invitrogen) to create p558 and p559, respectively. The inserts from p558 and p559 were excised by digestion with *Xho*I and *Hin*dIII and gel purified, and 10 µg of each was used to transform wt-*ΔakuA* protoplasts as previously described [15]. Loss of the *ireA* gene in phleomycin-resistant monoconidial transformants was confirmed by genomic Southern blot analysis, as described in the Results section.

The Δ*ireA* mutant was complemented by introducing the *ireA* gene into the Δ*ireA* mutant as an ectopic transgene. The *ireA* gene, including 550 bp upstream of the ATG start site was PCR-amplified from wt genomic DNA using primers 647 and 650 and cloned into pCR-Blunt II-TOPO (Invitrogen) to generate plasmid 564. Ten micrograms of p564 was then linearized with *Not*I and cotransformed into Δ*ireA* protoplasts with 1 µg of a plasmid containing the hygromycin resistance cassette (p373). Successful reconstitution of the *ireA* gene was confirmed in hygromycin-resistant monoconidial transformants by genomic Southern blot analysis (data not shown).

Disruption of the endoribonuclease domain of *ireA* was accomplished by deleting the nucleotide sequences encoding amino acids 1076–1085 using the Quickchange site-directed mutagenesis system (Stratagene). The complete *ireA* gene, together with 550 bp of promoter sequence, was PCR-amplified from wt genomic DNA and cloned into pCR-Blunt II-TOPO (Invitrogen) to generate plasmid 564. Next, p564 was used as a template for site-directed mutagenesis using the mutagenic oligonucleotides 701 and 702, according to the manufacturer's recommendations. Sequence analysis of the resulting plasmid (p596) confirmed the accuracy of the deletion. The *ireA* ^Δ10^ strain was constructed by linearizing 10 µg of p596 with *Not*I and co-transforming the plasmid into Δ*ireA* protoplasts together with 1 µg of p373, containing the hygromycin resistance cassette. Successful re-integration of the *ireA* ^Δ10^ allele into the *ireA* locus was confirmed by genomic Southern blot analysis and PCR sequencing on hygromycin-resistant monoconidial isolates.

The Δ*ireA*::*hacA* ^i^ strain was constructed by introducing the induced form of the *hacA* cDNA, *hacA* ^i^, into the background of the Δ*ireA* mutant. The *hacA* ^i^ cDNA was PCR amplified from reverse-transcribed cDNA using primer 493 (located 15 bp upstream of the ATG) and primer 572 (located 145 bp downstream of the *hacA* ^i^ stop codon). The PCR product was then cloned into pCR-Blunt II-TOPO (Invitrogen) to generate p576. The *hacA* ^i^ insert was excised from p576 by *Xba*I-*Sac*I digestion and inserted downstream of the constitutive *gpdA* promoter (P*gpdA*) using the same restriction sites to create p614. The P*gpdA*-*hacA* ^i^ cassette was excised from p614 with *Hind*III and *Sac*I and 10 µg was transformed into Δ*ireA* protoplasts and incubated at 37°C. Since the Δ*ireA* mutant is growth impaired at this temperature, colonies that appeared on the transformed plates before they started to appear on the untransformed Δ*ireA* control plates were transferred to fresh medium and ectopic integration of the *hacA* ^i^ expression cassette was confirmed by genomic Southern blot analysis and PCR.

### Analysis of *hacA* Splicing by RT-PCR

Overnight cultures of *A. fumigatus* were treated with 1 mM DTT for 1 h prior to extraction of total RNA. The RNA was prepared by crushing the mycelium in liquid nitrogen and resuspending in TRI reagent LS (Molecular Research Center, Cincinnati, OH). One microgram of the total RNA was reverse-transcribed with AMV reverse transcriptase using oligonucleotide 718 as the primer. The first-strand cDNA was then used as a template for PCR using primers 717 and 718, which flank the unconventional intron in the *hacA*u sequence. The PCR products were fractionated under denaturing conditions to remove hybrids between spliced and unspliced single-stranded DNA that can arise during PCR amplification (12% acrylamide/7M urea gel in 1X TBE) [56]. The samples were heated to 95°C for 5 min in RNA loading buffer (formamide-EDTA) prior to loading. The PCR products were stained with SYBR green II and fluorescence was quantified on a Personal Molecular Imager (PMI, Bio-Rad Laboratories, Hercules CA) using Quantity One and Image Lab software.

For validation of differentially expressed genes by qPCR, reverse transcription was performed using the SuperScript First-Strand Synthesis System (cat. no. 11904-018, Invitrogen) using an oligo-(dT)_18_ primer or an 18S rRNA-specific primer (primer #713) together with 1 µg of total RNA as template. The qPCR reaction was performed using the SYBR GreenER qPCR Super Mix (cat. no. 11762-100, Invitrogen) according to the manufacturer's protocol, using primer sets for the relevant target gene (Figure S9). The reactions were analyzed using a Smart Cycler II (Cepheid) with a standard two-step cycling program of 40 cycles at 95°C for 15 s and 60°C for 1 min; specificity and primer dimer formations were monitored using a melting curve. The Ct values were obtained using smart cycler software (v 2.0) and the relative changes in gene expression were calculated using the comparative Ct method, using 18S rRNA as the endogenous control and wt as the reference sample.

### Microarray Analysis

Cultures were inoculated with 5×10^6^ conidia in 5 ml of YG medium (0.5% yeast extract, 2% glucose) and incubated for 16 h with shaking at 37°C. Where indicated, the UPR was induced by treating 16 h-cultures with 1 mM dithiothreitol (DTT) or 10 µg/ml tunicamycin for 1 h. Total RNA was extracted by crushing the mycelium in liquid nitrogen and resuspending in TRI reagent LS (Molecular Research Center, Cincinnati, OH). The RNA labeling reactions and hybridizations were performed as described in the J. Craig Venter Institute (JCVI) standard operating procedure (http://pfgrc.jcvi.org/index.php/microarray/protocols.html) and transcriptional profiles were generated by interrogating the Af293 DNA amplicon microarray containing 9,516 genes [57]. Each gene was present in triplicate on the array, and all hybridizations were repeated in dye swap experiments. The data for each gene were averaged from the triplicate genes on each array and the duplicate dye swap experiment (a total of six readings for each gene) and the gene expression ratios were log_2_-transformed. Datasets were limited to genes that showed ≥1.5-fold change (log2-value of ±0.585). Functional annotation of genes present within the dataset was analyzed using BLAST2GO suite (PMID: 18445632) with standard settings (score alpha value set at 0.6). Gene Ontology Term Enrichment was performed using AmiGO Term Enrichment [58]. KEGG pathways associated with *Aspergillus fumigatus* were downloaded from the KEGG database [59]. Statistical significance of over-represented KEGG pathways was assessed using Fisher's exact test followed by correction using the Bonferroni method; a cutoff value of P<0.05 was assigned for statistical significance. Hierarchical clustering was performed using Cluster 3.0 [60] and the cluster tree was visualized using JAVA Treeview [61]. Microarray data was validated by demonstrating increased expression of known UPR target genes following DTT and Tm treatment (Table 1), by confirming expected phenotypic changes that correspond to specific changes in gene expression (Figure 10 and Figure 13) and by qPCR analysis of a subset of genes (Figure S10).

### Antifungal Susceptibility

Susceptibility to azole drugs was determined in broth culture using the Sensititre YeastOne kit (TREK Diagnostic Systems). The assay was performed according to the manufacturer's recommendations, with the exception of using *Aspergillus* minimal medium and an incubation temperature of 30°C to minimize the difference in growth rate between the wt and mutant strains. The minimal inhibitory concentration (MIC) is the lowest antifungal concentration showing inhibition of growth as indicated by the absence of a color change.

### Cell Wall Analysis

Mycelial cell wall fractionation was performed according to the method of Fontaine *et al*. [62], with slight modification. Briefly, the strains were grown in liquid YPD medium at 30°C with gentle shaking (150 rpm). After 24 h of growth, the mycelia were collected by filtration, washed extensively with water and disrupted in 50 mL Falcon tubes using the FastPrep-24 instrument (MP Biomedicals, Solon, United States). Disruptions were performed using 1 mm glass beads at 4°C, 6 m/s for one minute each, twice. The disrupted mycelial suspensions were centrifuged (3,000 g, 10 min) and the cell wall fractions (pellet) obtained was washed three times with water. Subsequent removal of proteins using SDS and β-mercaptoethanol, alkali-fractionation and estimation of the hexose composition by gas-liquid chromatography was performed as reported previously [15].

### Analysis of Ergosterol Content

A total of 1×10^7^ conidia were inoculated into 5 mL of YPD medium in a 50 mL conical tube and incubated at 30°C for 24 h, with gentle shaking (200 rpm). The biomass was washed with sterile distilled water and dried under vacuum. The dried mycelium was weighed prior to crushing under liquid nitrogen and then saponified in 1 mL of alcoholic KOH (3% KOH in ethanol). Stigmastanol was added as an internal standard and sterols were extracted into petroleum ether (hexane). The sterol concentrations were analyzed by gas chromatography using a known ratio of ergosterol and stigmastanol as the external standard. Values are presented as µg ergosterol per mg dry weight.

### Mouse Model of Invasive Aspergillosis

Conidia were harvested from OSM plates and resuspended in sterile saline. Groups of 12 CF-1 out-bred female mice (22 – 32 g, 6–8 weeks of age) were immunosuppressed with a single dose of triamcinolone acetonide (40 mg kg-1 of body weight) injected subcutaneously on day -1. The mice were anesthetized with 3.5% isofluorane and inoculated intranasally with 2×10^6^ conidia in a 20 µl suspension of saline. Survival was monitored for 2 weeks and persistence of the infection was assessed by plating the lungs of surviving mice onto inhibitory mold agar (IMA). Statistical significance of the mortality curve was assessed by Kruskal-Wallis ANOVA using Sigma Stat 3.5. A p-value of <0.001 was considered statistically significant.

For histopathological analysis of lung tissue, mice were infected as described above and sacrificed on days 1 and 3 post-infection. The lungs were fixed by inflation with 4% phosphate-buffered paraformaldehyde, dehydrated and embedded in paraffin, sectioned at 5 µm, and stained with hematoxylin and eosin (H&E) or Grocott methenamine silver (GMS). Microscopic examinations were performed on an Olympus BH-2 microscope and imaging system using Spot software version 4.6.

### Ethics Statement

Animal experiments were carried out in strict accordance with the Guide for the Care and Use of Laboratory Animals, the Public Health Service Policy on the Humane Care and Use of Laboratory Animals and all U.S. Animal Welfare Act Regulations. The experiments were approved by the Institutional Animal Care and Use Committee of the University of Cincinnati (protocol # 06-01-03-02). All efforts were made to minimize animal suffering.

### Genbank Accession Numbers for Genes in This Study

*A. fumigatus* IreA annotated in Genbank (XP_749922), *A. fumigatus* IreA cDNA sequenced in this study (JN653078), *A. fumigatus hacA* ^i^ (EU877964), *A. fumigatus hacA* (XM_743634).

## Supporting Information

Figure S1 **Multiple sequence alignment.** A schematic representation of the predicted domains in the IreA protein is shown at the top: signal peptide (SP), lumenal domain, transmembrane domain (TM), kinase domain and kinase extension nuclease (KEN) domain. A multiple sequence alignment of the protein kinase and KEN domains of Ire1 sequences is shown below: Tree (*Trichoderma reesei*), Afum (*A. fumigatus*), Aory (*Aspergillus oryzae*), Hsap (*Homo sapiens*), Scer (*S. cerevisiae*). The predicted KEN domain is underlined and the 10 amino deletion in the *ireA* ^Δ10^ mutant is indicated by the asterisk.
(TIF)

Figure S2 **Disruption of the ireA gene.** The *ireA* gene was deleted by replacing the entire coding region (open arrow) with the phleomycin resistance cassette (phleo). The flanking regions used to direct homologous recombination are indicated by the shaded boxes. Southern blot analysis of *Kpn*I/*Sma*I–digested genomic DNA using Probe A (flanking region) identified the predicted 10.3 kb wt band, which was truncated to 4.6 kb in the Δ*ireA* mutant. A second probe (probe B) derived from the *ireA* open reading frame was used to confirm the deletion and to demonstrate reconstitution in the complemented (C') strain (*Spe*I/*Bam*HI digest).
(TIF)

Figure S3 **Loss of ireA increases sensitivity to ER stress.** Equal numbers of conidia from the indicated strains were inoculated into each well of a multi-well plate containing YPD agar supplemented with the indicated concentrations of tunicamycin and incubated for 96 h at 30°C.
(TIF)

Figure S4 **Gene ontology enrichment table for the basal UPR.** Enrichment of functional annotations among genes with decreased abundance in Δ*hacA* and Δ*ireA* under standard laboratory culture conditions.
(DOC)

Figure S5 **Confirmation of the avirulence of** ***ΔireA*** **.** Groups of 12 CF-1 outbred mice were immunosuppressed with triamcinolone acetonide and infected intranasally with 2×10^6^ conidia from the wt, Δ*ireA* or the complemented (C') strains on day 0. Mortality was monitored for 12 days.
(TIF)

Figure S6 **Histopathology of infected lung tissue on days 1 and 3 post-infection.** Mice infected as described in Figure S5 were sacrificed on days 1 and 3 post-infection. The lungs were sectioned at 5 µm and stained with hematoxylin and eosin (H&E) or Grocott methenamine silver (GMS). Microscopic examinations were performed on an Olympus BH-2 microscope and imaging system using Spot software version 4.6. A high-power image of the Δ*ireA*-inoculated lungs reveals that the fungus could initiate germination in the host environment. Scale bar represents 100 µm.
(TIF)

Figure S7 **Hierarchical clustering of genes in the ergosterol biosynthetic pathway.** Genes were clustered by average linkage method using Gene Cluster 3.0 and visualized using Treeview. The figure shows that the decrease in abundance of transcripts related to steroid biosynthesis in Δ*hacA* and Δ*ireA* form four distinct groups. The greatest change in expression levels was for ERG11 (CYP51A).
(TIF)

Figure S8 **Strains used in this study.**
(DOC)

Figure S9 **Primers used in this study.**
(DOC)

Figure S10 **Validation of differentially expressed genes by qPCR.**
(DOC)

Figure S11 **Complete dataset.**
(XLS)
