# Supplementary figures and images for: HacA-Independent Functions of the ER Stress Sensor IreA Synergize with the Canonical UPR to Influence Virulence Traits in Aspergillus fumigatus
Source: PLoS Pathog. 2011 Oct 20;7(10):e1002330. doi: 10.1371/journal.ppat.1002330 (PMC3197630; doi:10.1371/journal.ppat.1002330)

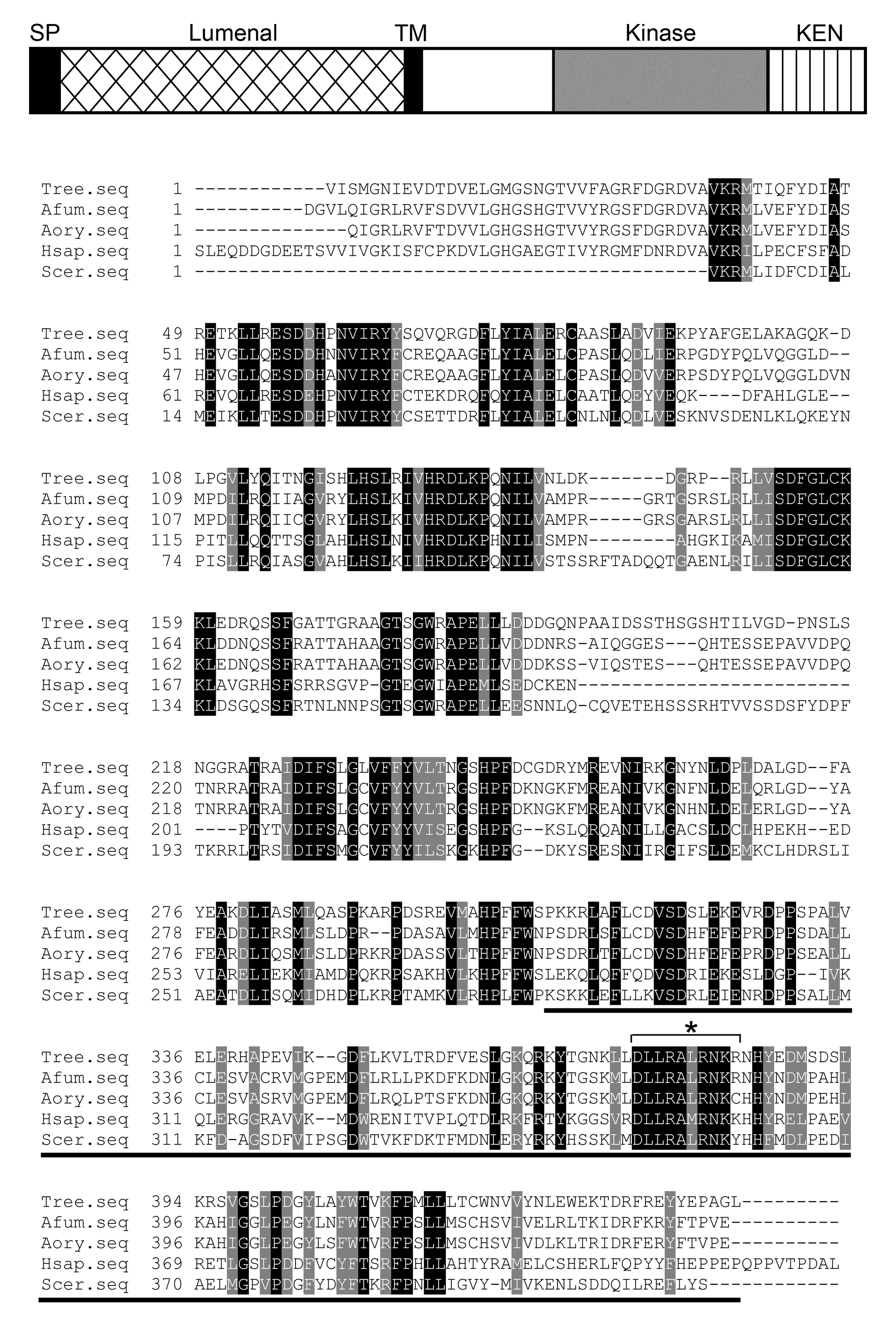

Supplement: Figure S1 — Multiple sequence alignment. A schematic representation of the predicted domains in the IreA protein is shown at the top: signal peptide (SP), lumenal domain, transmembrane domain (TM), kinase domain and kinase extension nuclease (KEN) domain. A multiple sequence alignment of the protein kinase and KEN domains of Ire1 sequences is shown below: Tree (Trichoderma reesei), Afum (A. fumigatus), Aory (Aspergillus oryzae), Hsap (Homo sapiens), Scer (S. cerevisiae). The predicted KEN domain is underlined and the 10 amino deletion in the ireA Δ10 mutant is indicated by the asterisk. (TIF) [file ppat.1002330.s001.tif]

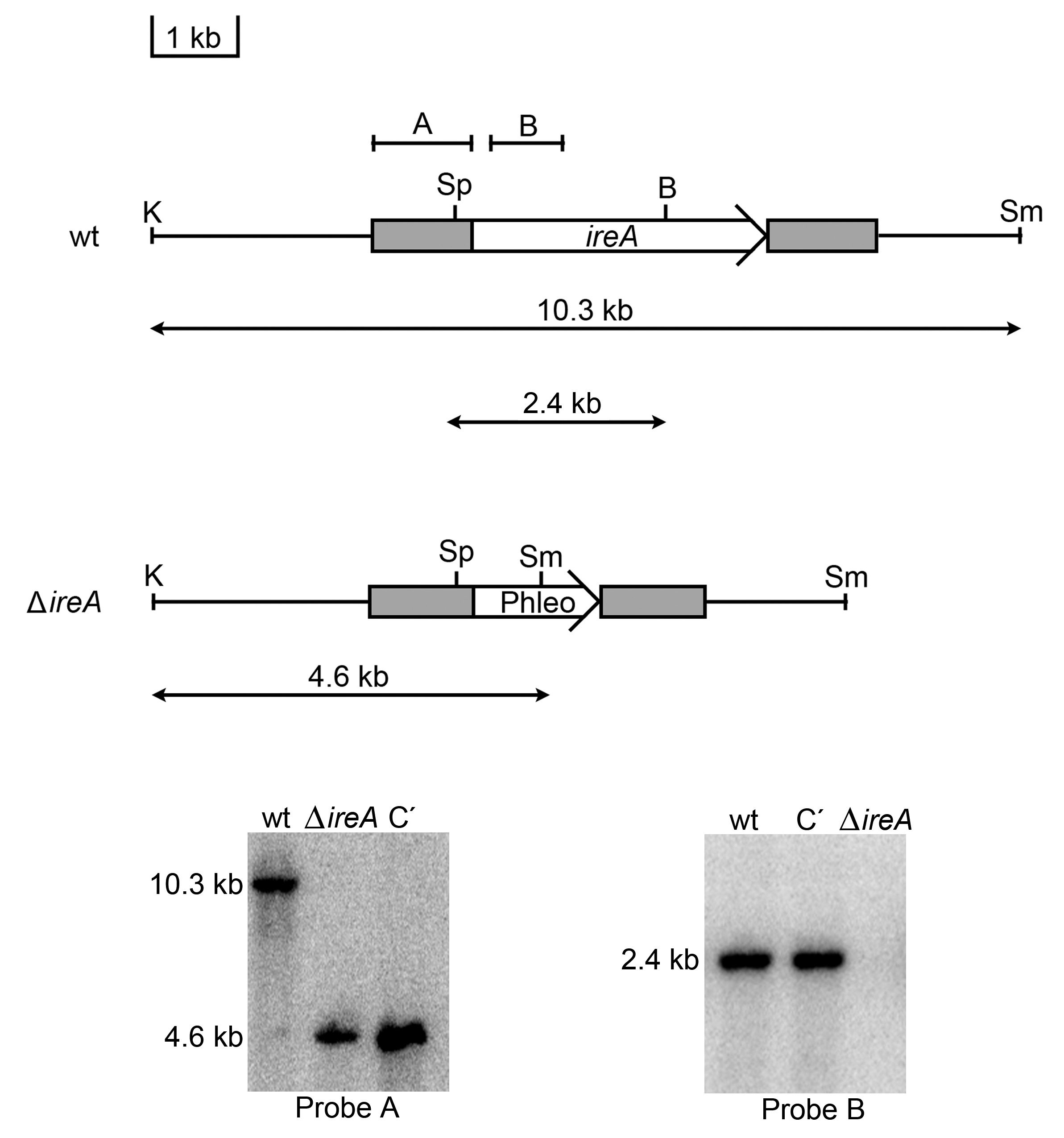

Supplement: Figure S2 — Disruption of the ireA gene. The ireA gene was deleted by replacing the entire coding region (open arrow) with the phleomycin resistance cassette (phleo). The flanking regions used to direct homologous recombination are indicated by the shaded boxes. Southern blot analysis of KpnI/SmaI–digested genomic DNA using Probe A (flanking region) identified the predicted 10.3 kb wt band, which was truncated to 4.6 kb in the ΔireA mutant. A second probe (probe B) derived from the ireA open reading frame was used to confirm the deletion and to demonstrate reconstitution in the complemented (C') strain (SpeI/BamHI digest). (TIF) [file ppat.1002330.s002.tif]

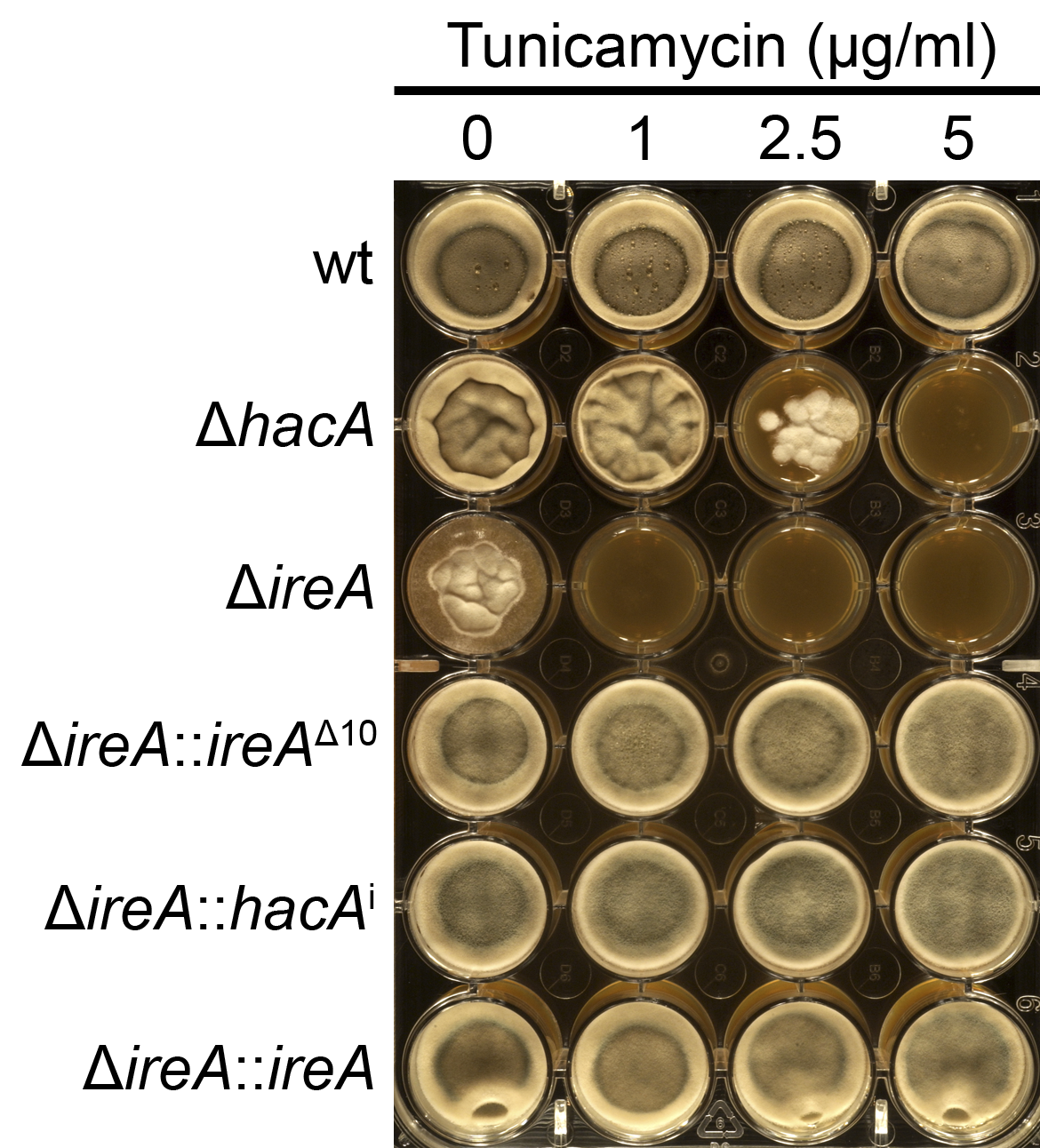

Supplement: Figure S3 — Loss of ireA increases sensitivity to ER stress. Equal numbers of conidia from the indicated strains were inoculated into each well of a multi-well plate containing YPD agar supplemented with the indicated concentrations of tunicamycin and incubated for 96 h at 30°C. (TIF) [file ppat.1002330.s003.tif]

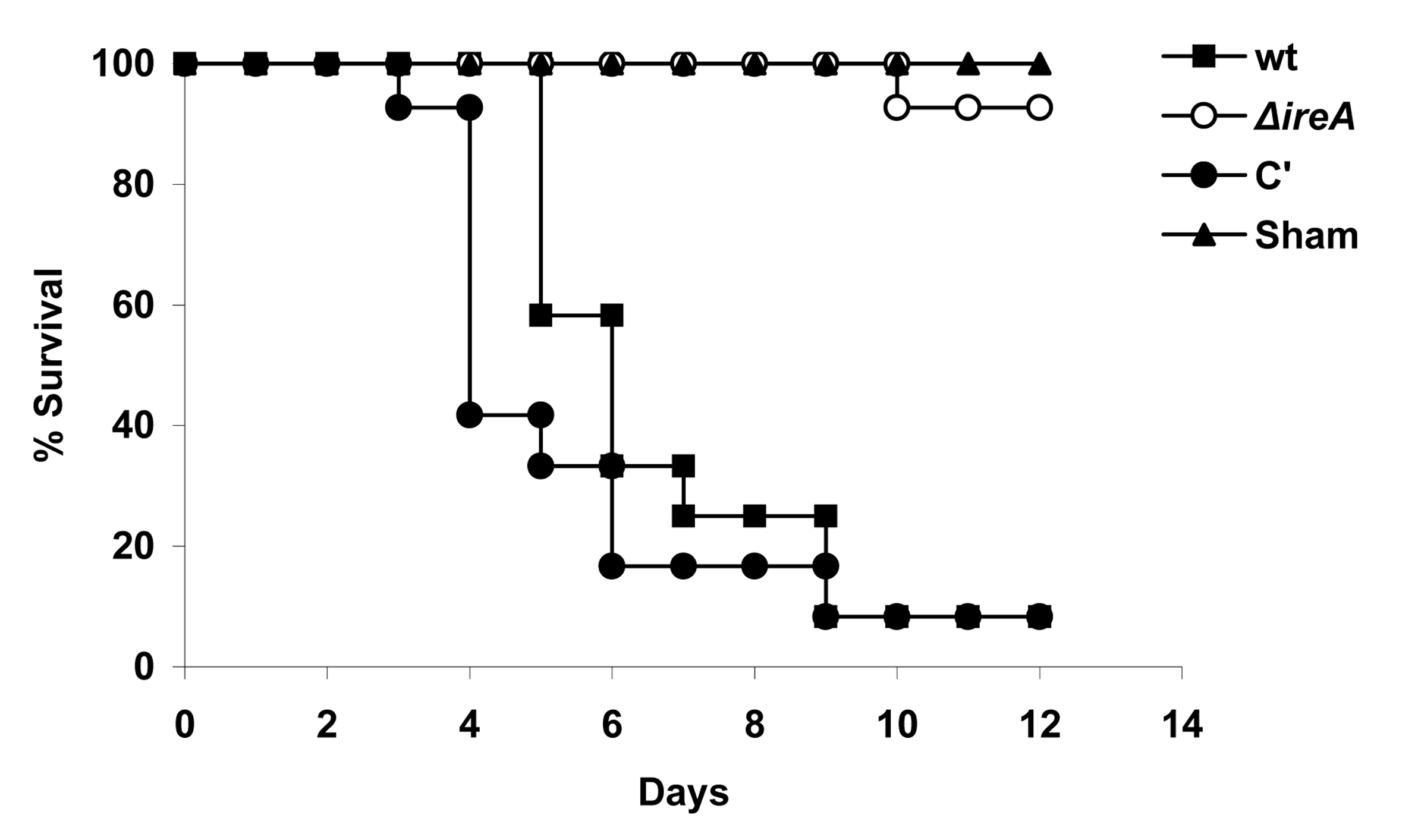

Supplement: Figure S5 — Confirmation of the avirulence of ΔireA . Groups of 12 CF-1 outbred mice were immunosuppressed with triamcinolone acetonide and infected intranasally with 2×10^6^ conidia from the wt, ΔireA or the complemented (C') strains on day 0. Mortality was monitored for 12 days. (TIF) [file ppat.1002330.s005.tif]

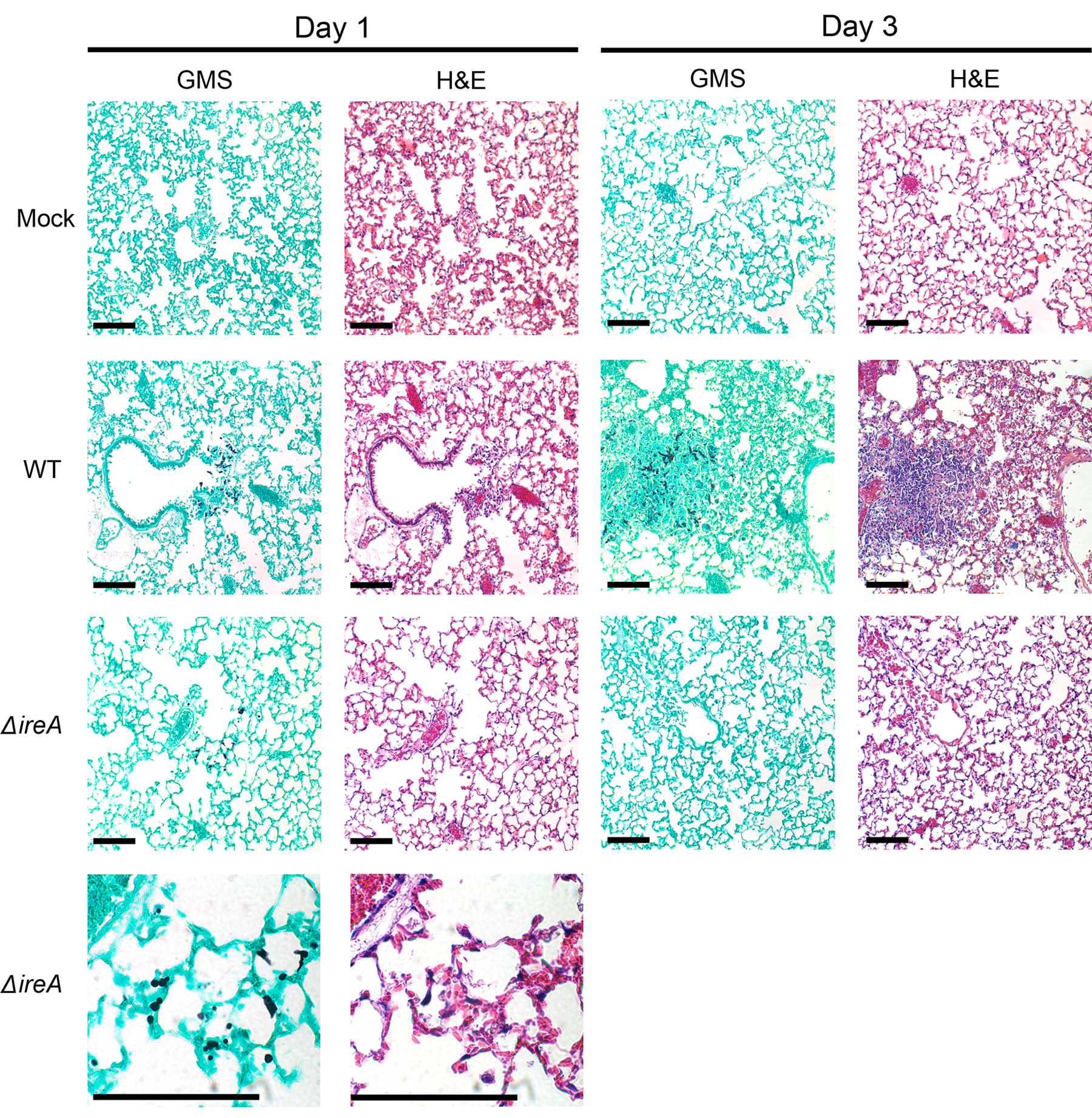

Supplement: Figure S6 — Histopathology of infected lung tissue on days 1 and 3 post-infection. Mice infected as described in Figure S5 were sacrificed on days 1 and 3 post-infection. The lungs were sectioned at 5 µm and stained with hematoxylin and eosin (H&E) or Grocott methenamine silver (GMS). Microscopic examinations were performed on an Olympus BH-2 microscope and imaging system using Spot software version 4.6. A high-power image of the ΔireA-inoculated lungs reveals that the fungus could initiate germination in the host environment. Scale bar represents 100 µm. (TIF) [file ppat.1002330.s006.tif]

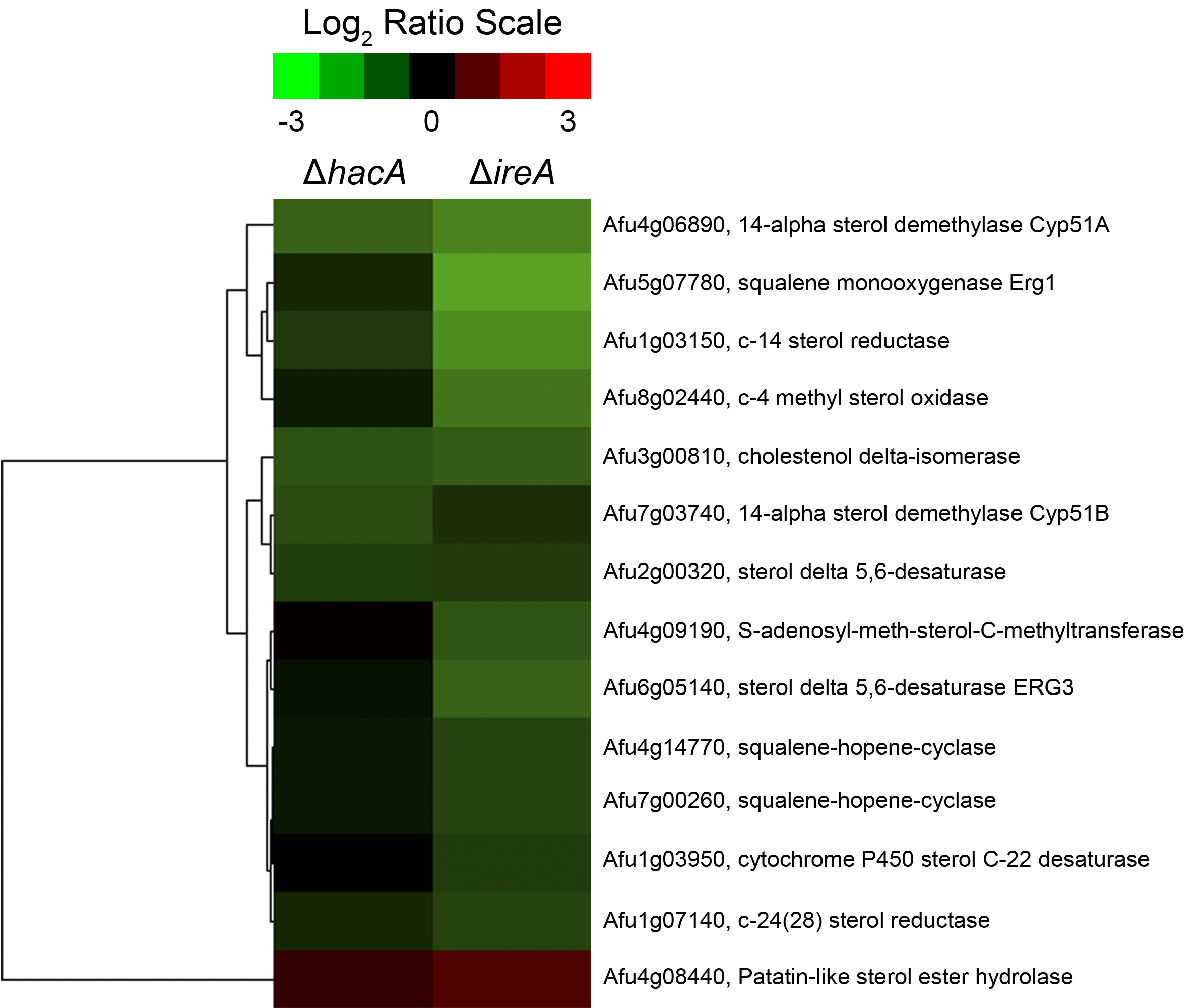

Supplement: Figure S7 — Hierarchical clustering of genes in the ergosterol biosynthetic pathway. Genes were clustered by average linkage method using Gene Cluster 3.0 and visualized using Treeview. The figure shows that the decrease in abundance of transcripts related to steroid biosynthesis in ΔhacA and ΔireA form four distinct groups. The greatest change in expression levels was for ERG11 (CYP51A). (TIF) [file ppat.1002330.s007.tif]
