# Supplementary material for: HacA-Independent Functions of the ER Stress Sensor IreA Synergize with the Canonical UPR to Influence Virulence Traits in Aspergillus fumigatus
Source: PLoS Pathog. 2011 Oct 20;7(10):e1002330. doi: 10.1371/journal.ppat.1002330 (PMC3197630; doi:10.1371/journal.ppat.1002330)
Supplement: Figure S4 — Gene ontology enrichment table for the basal UPR. Enrichment of functional annotations among genes with decreased abundance in ΔhacA and ΔireA under standard laboratory culture conditions. (DOC) [file ppat.1002330.s004.doc]

Figure S4. Enrichment of functional annotations among genes that show decreased mRNA abundance in the Δ*hacA* and Δ*ireA* mutants under standard growth conditions.

| **GO ID** | **GO term** | **p-value** |
| --- | --- | --- |
| GO:0016491^*^ | Oxidoreductase activity | 2.85 x 10^-5^ |
| GO:0070469**^§^** | Respiratory chain | 2.76 x 10^-3^ |
| GO:0005746**^§^** | Mitochondrial respiratory chain | 2.76 x 10^-3^ |
| GO:0005751**^§^** | Mitochondrial respiratory chain complex IV | 3.77 x 10^-3^ |
| GO:0045277**^§^** | Respiratory chain complex IV | 3.77 x 10^-3^ |
| GO:0016676^*^ | Oxidoreductase activity, acting on a heme group of donors, oxygen as acceptor | 3.77 x 10^-3^ |
| GO:0016675^*^ | Oxidoreductase activity, acting on a heme group of donors | 3.77 x 10^-3^ |
| GO:0015002^*^ | Heme-copper terminal oxidase activity | 3.77 x 10^-3^ |
| GO:0004129^*^ | Cytochrome-c oxidase activity | 3.77 x 10^-3^ |

* Molecular function in the parent GO category. **^§^**Cellular component in the parent GO category.
