# Supplementary material for: HacA-Independent Functions of the ER Stress Sensor IreA Synergize with the Canonical UPR to Influence Virulence Traits in Aspergillus fumigatus
Source: PLoS Pathog. 2011 Oct 20;7(10):e1002330. doi: 10.1371/journal.ppat.1002330 (PMC3197630; doi:10.1371/journal.ppat.1002330)
Supplement: Figure S8 — Strains used in this study. (DOC) [file ppat.1002330.s008.doc]

**Figure S8.** *A. fumigatus* strains used in this study

| **Strain** | **Genotype** | **Source** |
| --- | --- | --- |
| wt (AfS28) | Δ*akuA*::*ptrA* | Sven Krappmann |
| Δ*hacA* | Δ*akuA*::*ptrA*, Δ*hacA*::*hph* | This study |
| Δ*ireA* | Δ*akuA*::*ptrA*, Δ*ireA*::*ble* | This study |
| Δ*ireA*::*ireA*^Δ10^ | Δ*akuA*::*ptrA*, Δ*ireA*::*ireA*^Δ10^ | This study |
| Δ*ireA*::*hacA*^i^ | Δ*akuA*::*ptrA*, Δ*ireA*::*ble, (P_gpdA_-hacA*^i^*)* | This study |
| Δ*ireA*::*ireA* | Δ*akuA*::*ptrA*, Δ*ireA*::*ireA* | This study |
