# Supplementary material for: HacA-Independent Functions of the ER Stress Sensor IreA Synergize with the Canonical UPR to Influence Virulence Traits in Aspergillus fumigatus
Source: PLoS Pathog. 2011 Oct 20;7(10):e1002330. doi: 10.1371/journal.ppat.1002330 (PMC3197630; doi:10.1371/journal.ppat.1002330)
Supplement: Figure S9 — Primers used in this study. (DOC) [file ppat.1002330.s009.doc]

**Figure S9**. PCR primers used in this study. M13 sequences used for overlap PCR are underlined

| **Primer** | **Gene** | **Sequence (5′-3′)** | **Purpose** |
| --- | --- | --- | --- |
| 398 | *ble* | CGCCAGGGTTTTCCCAGTCACGACAAGTGGAAAGGCTGGTGTGC | *ireA* deletion |
| 408 | *ble* | TGCTCGCCGATCTCGGTCAT | *ireA* deletion |
| 409 | *ble* | AGCGGATAACAATTTCACACAGGATTAAAGCCTTCGAGCGTCC | *ireA* deletion |
| 410 | *ble* | GACAAGGTCGTTGCGTCAGTC | *ireA* deletion |
| 529 | *ireA* | GGTTGCTACACCTATTCAGG | *ireA* deletion probe A |
| 530 | *ireA* | GTCGTGACTGGGAAAACCCTGGCGGCATGAGGCTTTGCAGGTAA | *ireA* deletion probe A |
| 531 | *ireA* | TCCTGTGTGAAATTGTTATCCGCTGGTTGAGTAGGCTAGGTCTT | *ireA* deletion |
| 532 | *ireA* | CTGAAAACGACTGAGACTCC | *ireA* deletion |
| 492 | *hacA* | TGCGATAGACGCTGGAGAAG | *hacA* probe |
| 493 | *hacA* | CATCACGCCTACGAAATGGA | *hacA* probe |
| 534 | *ireA* | ACACAGCGTTCTTTCGACCA | *ireA* probe B |
| 535 | *ireA* | CCATTCGGAGTACTTCAACG | *ireA* probe B |
| 647 | *ireA* | CGACGTTGTAGCTTTTCACC | *ireA* complementation |
| 651 | *ireA* | GAAGCATCTCGATCACGGTC | *ireA* complementation |
| 648 | *ireA* | AAACCGAAACGAGACAGGAG | *ireA* cDNA |
| 649 | *ireA* | TCAAGTCTCGGTGCACAATC | *ireA* cDNA |
| 701 | *ireA* | CGGGCTCGAAGATGTTGGATCTCTACAACGACATGCCGGCACATC | *ireA*^Δ10^ cDNA |
| 702 | *ireA* | GATGTGCCGGCATGTCGTTGTAGAGATCCAACATCTTCGAGCCCG | *ireA*^Δ10^ cDNA |
| 493 | *ireA* | CATCACGCCTACGAAATGGA | *hacA*^i^ cDNA |
| 572 | *ireA* | TCGCTCGAATTTCGCGAAGA | *hacA*^i^ cDNA |
| 713 | *Af18SrRNA-R* | TGAGCCGATAGTCCCCCTAA | qPCR of 18S rRNA |
| 714 | *AF18sRNAprimerF-2* | GACTCAACACGGGGAAACTC | qPCR of 18S rRNA |
| 717 | *hacA* | TCCATTTCCCACTCCCTCA | *hacA* intron PCR |
| 718 | *hacA* | GGATGAATCACCGTTGAATAGG | *hacA* intron PCR |
| 778 | *Afu3g13670* | ACACGCTGTACTGGATTGGCTACA | qPCR of siderochrome-iron transporter, putative |
| 779 | *Afu3g13670* | GAATGCGTAGGCAAATGCCCGATT | qPCR of siderochrome-iron transporter, putative |
| 780 | *Afu8g01670* | TGGAGTCCATGGGTTTCAAGACCT | qPCR of bifunctional catalase-peroxidase Cat2 |
| 781 | *Afu8g01670* | TGTGTGACTCCTCGTCGGCAATAA | qPCR of bifunctional catalase-peroxidase Cat2 |
| 782 | *Afu3g13690* | ATCGACCACGAGATTGCCTCGAAT | qPCR of pyoverdine chromophore biosynthetic protein, putative |
| 783 | *Afu3g13690* | ACGCATCACCTCGTATCAGCATCT | qPCR of pyoverdine chromophore biosynthetic protein, putative |
| 784 | *Afu1g07480* | ACGCCTGTGATAGACATGATGCGA | qPCR of coproporphyrinogen III oxidase, putative |
| 785 | *Afu1g07480* | AAGAAGATACCGCCAACACCACGA | qPCR of coproporphyrinogen III oxidase, putative |
| 794 | *Afu4g07650* | GGCCCAAAGATCACAAGCAAGGTT | qPCR of peptidyl-prolyl cis-trans isomerase (CypB), putative |
| 795 | *Afu4g07650* | TGAAGCTCTTGATGACACGGTGGA | qPCR of peptidyl-prolyl cis-trans isomerase (CypB), putative |
|  |  |  |  |
