# Supplementary material for: HacA-Independent Functions of the ER Stress Sensor IreA Synergize with the Canonical UPR to Influence Virulence Traits in Aspergillus fumigatus
Source: PLoS Pathog. 2011 Oct 20;7(10):e1002330. doi: 10.1371/journal.ppat.1002330 (PMC3197630; doi:10.1371/journal.ppat.1002330)
Supplement: Figure S10 — Validation of differentially expressed genes by qPCR. (DOC) [file ppat.1002330.s010.doc]

Figure S10. Validation of differentially expressed genes by qPCR

| Locus | Common Name | N |
| --- | --- | --- |
| Afu3g13670* | Siderochrome-iron transporter, putative | 0.49 |
| Afu8g01670* | Bifunctional catalase-peroxidase Cat2 | 0.11 |
| Afu3g13690* | Pyoverdine chromophore biosynthetic protein, putative | 0.47 |
| Afu1g07480* | Coproporphyrinogen III oxidase, putative | 0.52 |
| Afu5g07780* | Squalene monooxygenase Erg1 | 0.59 |
| Afu4g06890* | 14-α sterol demethylase Cyp51A (Erg11A) | 0.82 |
| Afu4g07650^£^ | Peptidyl-prolyl cis-trans isomerase (CypB), putative | 1.21 |
| Afu2g03700^¥^ | HMG-CoA reductase | 3.26 |

N = relative changes in gene expression calculated as described in materials & methods

*Decreased abundance (>1.5-fold) in Δ*ireA* when compared with wt in microarray experiments

^£^No change (<1.5-fold) in Δ*ireA* when compared with wt in microarray experiments

^¥^Increased abundance (>1.5-fold) in Δ*ireA* when compared with wt in microarray experiments
